# Live cell imaging and proteomic profiling of endogenous NEAT1 lncRNA by CRISPR/Cas9-mediated knock-in

**DOI:** 10.1007/s13238-020-00706-w

**Published:** 2020-05-26

**Authors:** Bohong Chen, Shengcheng Deng, Tianyu Ge, Miaoman Ye, Jianping Yu, Song Lin, Wenbin Ma, Zhou Songyang

**Affiliations:** 1 Sun Yat-sen Memorial Hospital, Sun Yat-sen University; MOE Key Laboratory of Gene Function and Regulation and Guangzhou Key Laboratory of Healthy Aging Research, SYSU-BCM Joint Research Center, School of Life Sciences, Sun Yat-sen University, Guangzhou, 510275 China; 2 State Key Laboratory of Ophthalmology, Zhongshan Ophthalmic Center, Sun Yat-sen University, Guangzhou, 510060 China; 3 Marrs Mclean Department of Biochemistry and Molecular Biology, Baylor College of Medicine, One Baylor Plaza, Houston, TX 77030 USA

**Keywords:** CRISPR/Cas9 genome editing, endogenous lncRNA labeling, MS2-MCP, NEAT1, paraspeckle dynamics

## Abstract

In mammalian cells, long noncoding RNAs (lncRNAs) form complexes with proteins to execute various biological functions such as gene transcription, RNA processing and other signaling activities. However, methods to track endogenous lncRNA dynamics in live cells and screen for lncRNA interacting proteins are limited. Here, we report the development of CERTIS (CRISPR-mediated Endogenous lncRNA Tracking and Immunoprecipitation System) to visualize and isolate endogenous lncRNA, by precisely inserting a 24-repeat MS2 tag into the distal end of lncRNA locus through the CRISPR/Cas9 technology. In this study, we show that CERTIS effectively labeled the paraspeckle lncRNA NEAT1 without disturbing its physiological properties and could monitor the endogenous expression variation of NEAT1. In addition, CERTIS displayed superior performance on both short- and long-term tracking of NEAT1 dynamics in live cells. We found that NEAT1 and paraspeckles were sensitive to topoisomerase I specific inhibitors. Moreover, RNA Immunoprecipitation (RIP) of the MS2-tagged NEAT1 lncRNA successfully revealed several new protein components of paraspeckle. Our results support CERTIS as a tool suitable to track both spatial and temporal lncRNA regulation in live cells as well as study the lncRNA-protein interactomes.

**Electronic supplementary material:**

The online version of this article (10.1007/s13238-020-00706-w) contains supplementary material, which is available to authorized users.

## Introduction

Noncoding RNAs (ncRNAs) have been characterized as the dark matter of the human genome because they do not encode protein sequences despite taking up a major portion of our transcriptome (Derrien et al., 2012; Evans et al., 2016; Jandura and Krause, 2017). ncRNAs that longer than 200 nt are called long noncoding RNAs (lncRNAs) and are particularly interesting due to their important roles in a variety of cellular activities (Marchese et al., 2017; Kopp and Mendell, 2018; Yao et al., 2019). It is well documented that lncRNAs can fold into structural units and form complexes with proteins such as RNA binding proteins (RBPs) to regulate different functions, including gene imprinting, antiviral response, differentiation and development (Weinrich et al., 1997; Nguyen et al., 2001; Xing et al., 2017; Munschauer et al., 2018). One intrigue characteristic of lncRNAs is that they may be post-transcriptionally modified and bind different RBPs to regulate diverse biological functions. For instance, during developmental process or under stress, the localization and organization of lncRNA RNP are regulated spatially and temporally by RNA modification and changes of RNA binding proteins to adapt to their functions being performed (Yang et al., 2015; Chen, 2016). In other words, lncRNA RNP could be dynamic. Therefore, studying the spatiotemporal distribution and interactive proteomics of lncRNA within cells play central roles to unmask their biological functions and mechanisms during physiological and pathological processes.

Some of the lncRNAs can play an architectural role with RBPs in the assembly of subcellular structures, such as paraspeckle and nuclear speckle in the nucleus (Hutchinson et al., 2007), which are ideal models to illustrate the dynamical regulation of lncRNA-protein complexes. Paraspeckles are mammal-specific, membraneless RNA-protein nuclear bodies that built on a long, non-protein-coding RNA, nuclear-enriched abundant transcript 1 (NEAT1) and a series of core RNA-binding structural protein components (Sunwoo et al., 2008; Clemson et al., 2009; Sasaki et al., 2009; Fox et al., 2018). Since paraspeckles first described in 2002 as nuclear foci, the knowledge on paraspeckles is accumulating at a rapid rate (Fox et al., 2002; Naganuma et al., 2012). Existing research suggests that paraspeckles play a role in a variety of developmental and disease scenarios, including female reproduction, viral infection, ALS pathogenesis and cancer progression (Zhang et al., 2013; Nakagawa et al., 2014; Shelkovnikova et al., 2014; Standaert et al., 2014; Fujimoto et al., 2016; Lanzós et al., 2017; Wang et al., 2017). However, the functional studies are not as advanced. As yet, no distinct catalytic activity has been found to occur within paraspeckles, while it is commonly believed that the molecular basis of paraspeckle function mainly consequent on the sequestration of certain RNA species and paraspeckle protein components by the scaffold of NEAT1 lncRNA (Chen and Carmichael, 2009; Hirose et al., 2014; Wang et al., 2018).

However, study of such a highly dynamical lncRNA has been limited by a lack of efficient and versatile method that can both track endogenous lncRNA dynamics in live cells and identify lncRNA interacting proteins. Currently, methods based on RNA antisense nucleotide probe hybridization are the golden standard for studying lncRNA localization and interaction. Visualization of lncRNAs could be achieved with high specificity and signal-to-noise ratio (SNR) by RNA fluorescent in situ hybridization (RNA FISH) (Levsky and Singer, 2003; Itzkovitz and van Oudenaarden, 2011). Although RNA FISH is ideal for fixed samples, it is incompatible to track the subcellular lncRNA dynamics in live cells. Methods using biotinylated oligonucleotides complementary to the lncRNAs to identify lncRNA-interacting proteins including RAP and ChIRP (Cao et al., 2019). However, both of these methods are cross-linking and hybridization-based, which may disrupt lncRNA structure and are insufficient for the study of lncRNA with higher structure or RBP occupancy. Other latest RNA labeling systems such as RNA-targeting Cas9 (RCas9) and nuclease-inactive dCas13a (Nelles et al., 2016; Rau and Rentmeister, 2016; Cox et al., 2017), although both of them have shown satisfactory SNR on the cytoplasm mRNAs trafficking in live cells, neither of RCas9 and dCas13a has been developed as a routine method to identify the interaction proteins of labeled RNA until now. Given the intricate connection between lncRNA localization, RBP interactions and functions, there is appealing need to develop methods to visualize and purify endogenous lncRNA-RNPs.

The MS2-MCP system has been widely used for labeling exogenous RNA from yeast, zebrafish to higher organisms (Wu et al., 2012; Liu et al., 2015; Tutucci et al., 2018a, b), which enables efficient RNA immunoprecipitation and tracking of exogenous RNA with single-molecule sensitivity in live cells. MS2 is a stem-loop bacteriophage RNA that specifically binds to the MS2 coat protein (MCP) with high affinity, where multiple copies of MS2 (up to 24) can further improve the SNR as each MS2 is able to bind an MCP:FP dimer (Fusco et al., 2003). Moreover, MS2 tags have also been used to visualize endogenous RNA localization and trafficking in living yeast, mouse or human cells recently (Lionnet et al., 2011; Park et al., 2014; Tutucci et al., 2018a, b; Kim et al., 2019; Spille et al., 2019; Yang et al., 2019), demonstrating that the insertion of MS2 repeats would not disturb the spatiotemporal distribution of certain RNAs. However, considering the highly complicated human genome that can lead to unexpected off-targets and much lower efficiency of homologous recombination, precisely tagging the endogenous RNAs especially lncRNA with MS2 in human cells is still a challenging task.

Here, we develop a simple, efficient and scalable endogenous lncRNA labeling system called CERTIS (CRISPR-mediated Endogenous lncRNA Tracking and Immunoprecipitation System) in human cells. By combining CRISPR/Cas9-mediated gene knock-in (KI) and MS2-MCP RNA labeling system, it enables both live cell imaging and immunoprecipitation of endogenous lncRNA-protein complexes, as well as monitoring the expression level variation of lncRNA. First, CERTIS knocked in the MS2-repeat cassette in the distal end of lncRNA locus by CRISPR/Cas9-induced micro-homology mediated end joining (MMEJ). In order to enrich the KI cell population as well as monitor the lncRNA expression level, an IRES-GFP fragment was added after the MS2-repeat and driven by the endogenous promoter. Successfully KI cells were then enriched by consecutive GFP positive sorting before subjected to clonal isolation and genotyping. In case of the unexpected off-target insertions and aiming at recycling GFP for future multi-color labeling, another round of GFP negative selection could be further performed optionally by precise excision of the IRES-GFP fragment. After stably expressing MCP and its fusion fluorescent protein tags, visualization or affinity purification of targeted lncRNA-protein complex could be achieved using these cells. In this study, we show that CERTIS effectively labeled endogenous paraspeckle lncRNA NEAT1 without disturbing its physiologic properties and could be used to quantitatively monitor the expression variation of endogenous NEAT1. Moreover, CERTIS achieved superior performance in live cell imaging of both short- and long-term tracking of NEAT1 dynamics. Using this system, we were able to determine the effect of different classes of cellular stress on NEAT1 expression and dynamics. For example, we found that NEAT1 and paraspeckles were sensitive to topoisomerase I specific inhibitors and viral infection. In addition, we performed RNA Immunoprecipitation (RIP) of tagged NEAT1 lncRNA and identified several new protein components of paraspeckle. Overall, our results suggest that CERTIS is a tool suitable to track both spatial and temporal lncRNA dynamics in live cells as well as study the lncRNA-protein interactomes.

## Results

### Targeting the NEAT1 lncRNA through MMEJ-mediated CRISPR KI

**Figure 1:**
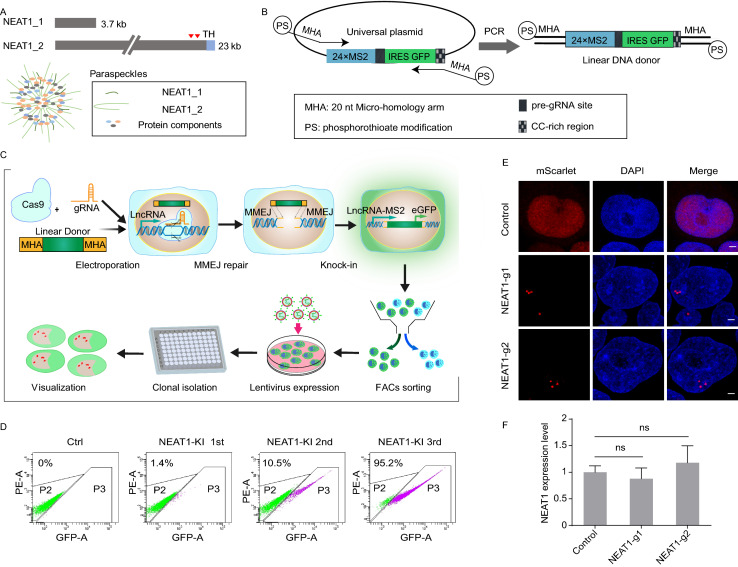
**Targeting endogenous NEAT1 lncRNA by MMEJ-mediated CRISPR knock-in**. (A) Schematic of NEAT1 isoforms and paraspeckle structure. NEAT1 contains two isoforms as indicated, and the longer isoform NEAT1_2 plays an architectural role in the assembly of paraspeckle with other RBPs. NEAT1 transcripts are radially arranged perpendicular to the longer axis of paraspeckles with the 5′ and 3′ ends of the transcripts facing outward. The red inverted triangles indicate the targeted site of the gRNA-1 and gRNA-2. TH indicates the triple helix structure of NEAT1_2. (B) Schematic of one-step generation of linear DNA donor for MMEJ-based CRISPR knock-in. The PCR primers contain 20 nt MHAs around the cleave site and 25-30 nt overlapping region of the universal template plasmid. PS indicates phosphorothioate modification between the last 5 nt at the 5′ end of PCR primers. The universal template of the plasmid mainly contains 24×MS2-IRES-GFP cassette, and the IRES-GFP fragment is flanked with a pre-gRNA site and a CC-rich region for further cleavage. (C) Workflow of CERTIS labeling in target lncRNA genomic locus. The workflow includes electroporation of Cas9 RNP and linear DNA donor, FACs enrichment of GFP positive cells, stable expression of tdMCP-mScarlet-3×Flag cassette, clonal isolation and identification of correctly KI clones. (D) Enrichment of NEAT1 KI cells by GFP FACs sorting. Representative images showed consecutive FACs sorting were proceeded to isolate the GFP positive cells. P3 was the gate for GFP positive selection. After three rounds of sorting, the cell population was nearly 100% GFP positive. (E) Visualization of endogenous NEAT1 (red) by lentiviral expression of tdMCP-mScarlet-3×Flag in both KI cell lines. Cells were fixed and stained with DAPI (blue). WT HEK293T cells stably expressing the tdMCP cassette were used as control. Scale bar: 2 μm. (F) Expression level of NEAT1 was measured by RT-qPCR in WT HEK293T and the two KI cell lines. No significant difference was detected in expression between WT and KI cell lines. ns, no significant difference, determined by two-tailed Student’s t test. The error bars represent standard deviation from three parallel experiments

The NEAT1 gene locus generates two noncoding isoforms in human: the 3.7 kb short NEAT1_1 and the 23 kb long NEAT1_2 (Fig. 1A). NEAT1_1 completely overlaps the 5′ end of NEAT1_2. Although both NEAT1 transcripts localize to the paraspeckle, NEAT1_2, but not NEAT1_1, play an architectural role in the formation of paraspeckles (Clemson et al., 2009). EM and super-resolution microscopy have been used to reveal that paraspeckles are composed of fused chains of spherical subcompartments, with NEAT1_2 arranged perpendicular to the longer axis of paraspeckles and its 5′ and 3′ ends facing outward (Hu et al., 2016; West et al., 2016). Thus, observation and manipulation of NEAT1_2 is a route to functional studies of paraspeckles, which needs to target the unique C terminal distal end of NEAT1_2 transcript. Unless otherwise specified, NEAT1 hitherto refers to the longer isoform. Considering the important functions and obscure regulatory mechanism of NEAT1, a new approach is now developed, aiming at labeling such a highly dynamical structural lncRNA.

Recently, CRISPR/Cas9 technology has been developed to edit and label genomic loci in the living cell due to its accuracy and efficiency on the recognition of DNA and RNA (Nelles et al., 2016; Shao et al., 2016; Qin et al., 2017; Tasan et al., 2018). CRISPR/Cas9-mediated gene KI usually employs donor templates with long homologous arms, ranging from dozens of nucleotides for single-stranded oligodeoxynucleotides (ssODNs) to several hundred base pairs for larger fragments. The process relies on homology-directed recombination (HDR), which occurs predominantly during late S/G_2_ phases of the cell cycle and has relatively low efficiency in human cells (Taleei and Nikjoo, 2013). Moreover, traditional HR method needs laborious construction of donor vectors with long homology arms, which impedes high-throughput applications. Interestingly, it was shown that donor templates with considerably shorter homologous arms (<20 nt) could mediate efficient and successful KI via the MMEJ pathway (Bae et al., 2014; Nakade et al., 2014; Kostyrko and Mermod, 2016). Unlike HDR, the alternative end-joining process MMEJ uses microhomology regions of 5–25 bp that flank a DSB to repair DNA and has high activity during the G_1_ and early S phases of the cell cycle. The short homology arms also make it easier to amplify templates and scale-up different loci targeting by one-step, cloning-free PCR.

To engineer the MS2-MCP system to the labeling of endogenous NEAT1, we designed and optimized the workflow for efficient CRISPR/Cas9 knock-in of MS2 repeats to the distal end of targeted site (Fig. 1C). First, we adopted a PCR-based strategy using primers that contain at their 5′ ends 20 nt micro-homology arms (MHAs) and a 25–30 nt overlapping region of the universal template plasmid, to exponential amplification of linear donor DNA. Therefore, we only need to replace the 20 nt MHAs at the 5′ end of the primers to prepare the DNA donor templates for scalable lncRNAs labeling. Notably, to avoid the rapid degradation of linear DNA fragment in the nucleus, the 5′ end of primers were thiophosphorylated to their last 5 nt (Fig. 1B). The universal plasmid template contains the 24×MS2-IRES-GFP cassette, where IRES-GFP is flanked by a nontarget pre-gRNA site and a CC-rich region (as PAM motifs). This design aims to minimize random off-target insertions or recycle the GFP for multi-color labeling as a further choice.

Two gRNAs were designed to target the distal end of NEAT1 respectively (Fig. 1A and Table S1). Following introduction of the donor template and Cas9 RNP into HEK293T, successful KI cells were enriched by consecutive several rounds of FACs sorting (Fig. 1D). mScarlet is a strong red fluorescence protein with superior anti-photobleaching properties (Bindels et al., 2017), which is ideal for long-term tracking of tagged proteins. When we expressed tdMCP-mScarlet-3×Flag in the two independent MS2 KI cell lines, speckle-like puncta could be both observed in the nucleus compared to the control cells (Fig. 1E), indicating that these are likely paraspeckles revealed by MCP-mScarlet expression. Then the cells were plated to 96-well-plate to isolate single cell clones. To further confirm the correct KI of MS2-repeat, genotyping PCR was carried out with junction PCR primers and followed by Sanger sequencing (Figs. S1, S7 and S8). The expected band size of PCR products for both NEAT1 KI cell lines revealed the successful and efficient insertion by MMEJ-based KI (NEAT1-g1: 19/19 and NEAT1-g2: 16/19). Sanger sequencing after TA cloning of the PCR products confirmed that most of the clones were precisely inserted into the expected loci. One clone from each gRNA KI was used for further analysis.

### Endogenous NEAT1 distally tagged with the MS2 cassette displays normal spatiotemporal distribution and function

**Figure 2:**
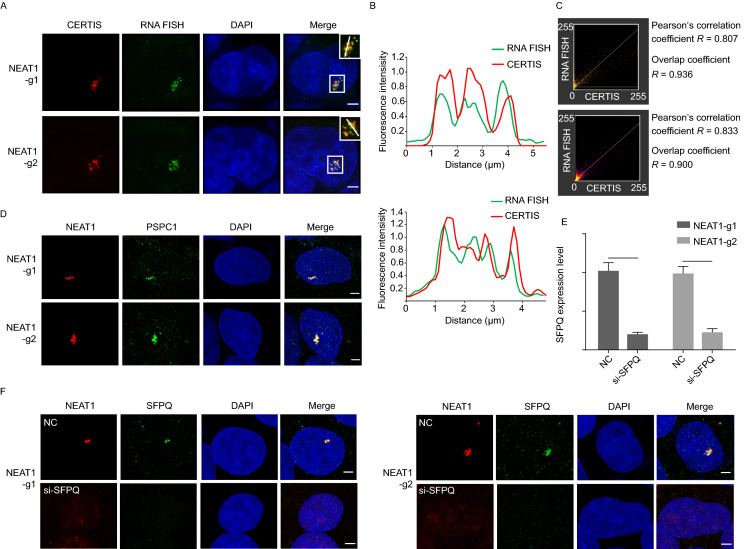
**Insertion of 24×MS2 cassette has no effect on the spatiotemporal distribution and function of endogenous NEAT1**. (A) Co-labeling of NEAT1 in CERTIS-labeled (red) cell lines with RNA FISH (green). The nucleus was stained by DAPI (blue). The insets show the magnified image of merged red and magenta channels of the boxed region. Magenta signal was defined as green. (B) Plot profiles along the white line in (A) inset. CERTIS-labeled NEAT1 co-localized well with RNA FISH probe in both NEAT1-g1 (Upper panel) and NEAT1-g2 (Lower panel) cell lines. (C) The scatter plots showed the intensity correlation of the two channels in NEAT1-g1 (upper panel) and NEAT1-g2 (lower panel) for each individual pixel. NEAT1-g1: Pearson’s correlation coefficient *R* = 0.807, Overlap coefficient *R* = 0.936. NEAT1-g2: Pearson’s correlation coefficient *R* = 0.833, Overlap coefficient *R* = 0.900. (D) Paraspeckle was immunostained with anti-PSPC1 antibody (green). The merged image showed that CERTIS-labeled NEAT1 (red) overlapped well with paraspeckle component protein. NEAT1 signals were primarily in paraspeckle foci but not in nucleoplasm. (E) Both NEAT1 KI cell lines were transfected with a scramble siRNA oligo (NC) or an oligo against SFPQ (si-SFPQ). The knock down efficiency was quantified by qPCR after 48 h transfection. GAPDH was used as the internal control, ***P < 0.001. (F) Paraspeckle was immunostained with anti-SFPQ antibody (green). SFPQ deficiency led to disaggregation of paraspeckle in both NEAT1 KI cell lines. The merged images showed that NEAT1 could not form puncta in the nucleus after SFPQ knock down. All scale bars are 2 μm

When we examined the expression level of NEAT1 in the two KI cell lines with parental HEK293T by RT-qPCR, we observed no significant difference between the cell lines (Fig. 1F). Next, we carried out RNA-FISH analysis using an Alexa Flour 647-labeled NEAT1 RNA probe that targets the middle region of NEAT1 (Fig. 2A and 2B). The super-resolution microscopy STEP revealed the mScarlet signals (red, endogenous NEAT1) overlapped well with signals from the FISH probe (defined as green). The scatter plots also demonstrated that the mScarlet channel colocalized well with FISH probe channel for each individual pixel, with both high Pearson’s correlation coefficient and overlap coefficient (Fig. 2C). Interestingly, less noise in the background was observed from CERTIS approach compared to FISH, probably because CERTIS avoids the cumbersome steps that might cause the residual non-specific binding of FISH probe.

To further evaluate the MS2-tagged NEAT1, we marked paraspeckles with antibodies that recognize paraspeckle marker proteins PSPC1 and SFPQ. Both proteins are essential components of paraspeckles and directly interact with NEAT1 (Fox et al., 2005; Lee et al., 2015). In fact, either of their deficiency can result in NEAT1 instability and eventual disaggregation of paraspeckles (Naganuma et al., 2012). We found extensive overlapping of mScarlet-labeled NEAT1 and Alexa Flour 647-labeled PSPC1 or SFPQ could be observed in both of MS2 KI cell lines (Fig. 2D and 2F). Furthermore, when we knocked down SFPQ by siRNA in the NEAT1-labeled cells, mScarlet signals appeared diffused in the nucleus with no clear puncta formation, consistent with paraspeckle disaggregation after SFPQ deficiency (Fig. 2E and 2F). Taken together, these results suggest that the MS2-tagged NEAT1 is able to correctly associate with protein binding partners and assemble paraspeckles, and that these cells are a good model system to study NEAT1 lncRNA and its binding proteins.

### CERTIS quantitatively monitors both spatial and temporal regulation of NEAT1 in live cells

**Figure 3:**
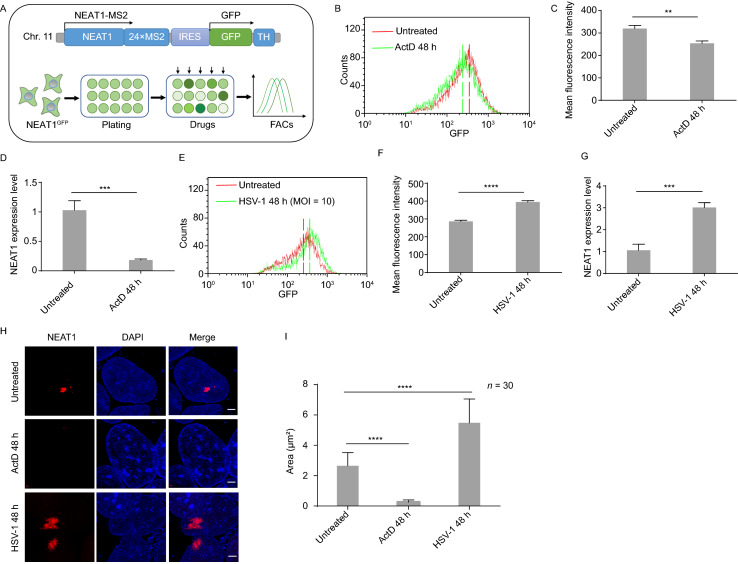
**CERTIS quantitatively monitors the expression variation of NEAT1 in live cells**. (A) Schematic demonstrated the CERTIS labeled NEAT1 and strategy for quantitatively monitoring NEAT1 regulators in live cells. NEAT1 was tagged with 24×MS2 and GFP expression was driven by the endogenous promoter of NEAT1. The GFP intensity variation indicates NEAT1 level regulation. (B) Histogram showed the expression change of NEAT1 after ActD treatment. GFP peak shifted to the left after 250 ng/mL ActD treatment for 48 h. Untreated cells were used as control. The dotted lines indicated the mean of GFP fluorescence intensity. (C) Mean fluorescence intensity (from (B)) significant decrease after ActD treatment for 48 h. (D) NEAT1 expression level with or without ActD treatment were quantified by RT-qPCR. (E) Histogram showed the expression level change of NEAT1 after HSV-1 infection. GFP peak shifted to the right after HSV-1 infection for 48 h. HSV-1 infection MOI = 10. (F) Mean fluorescence intensity (from E) significant increased after HSV-1 treatment. (G) NEAT1 expression levels with or without HSV-1 stimulation were quantified by RT-qPCR. (H) Fluorescent imaging of mScarlet signal at paraspeckles revealed the spatial regulations of NEAT1. The size of NEAT1 puncta (red) diminished after ActD treatment and increased after HSV-1 infection. Scale bar: 2 μm. (I) The mean area of paraspeckle foci within nucleus significant reduced after ActD treatment and enlarged after HSV-1 stimulation for 48 h compared to the untreated cells (*n* = 30). All error bars represent SD in triplicate experiments

Quantitatively reporting of lncRNA expression in live cells would aid our understanding of lncRNA function and its regulatory mechanisms, especially in the application of high-throughput gene inactivation or drug screening. Taking the advantage that CERTIS labeled NEAT1 was tagged by endogenous promoter-driven IRES-GFP, we next evaluated whether change of lncRNA level could be monitored by the GFP intensity through flow cytometry (Fig. 3A). Actinomycin D (ActD) could decrease NEAT1 expression level rapidly owing to its RNA synthesis inhibition capability (Fox et al., 2002). We treated the NEAT1-g1 KI cells with ActD for 48 h and then measured the expression of NEAT1 by flow cytometry. Compared to untreated cells, the GFP peak shifted to the left and mean fluorescence intensity significantly decreased after ActD treatment, indicating a diminishing of NEAT1 expression was detected (Fig. 3B and 3C). This was consistent with RT-qPCR quantification of NEAT1 level (Fig. 3D). Since herpes simplex virus 1 (HSV-1) infection has been reported that could enhance the expression of NEAT1 (Imamura et al., 2014), we next treated the cells with HSV-1 under the indicated MOI and determined the variation of GFP intensity. Indeed, an expected improvement of GFP level was observed in line with the NEAT1 expression after 48 h HSV-1 infection (Fig. 3E–G). Moreover, we found that the fluorescent imaging of mScarlet signal at paraspeckles could also truthfully monitor the spatial regulations of NEAT1. During ActD treatment, a diminishing of NEAT1 was observed, which disaggregated from the paraspeckle puncta and dispersed rapidly within the nucleus, while increasing both the number and total area of paraspeckles in response to HSV-1 infection (Fig. 3H and 3I). These results above demonstrated that both the spatial and temporal regulation of CERTIS labeled lncRNA could be faithfully measured in live cells, paving ways for potential high-throughput screening of lncRNA regulators.

### Tracking of NEAT1 and paraspeckles dynamics in response to different stimuli over time

**Figure 4:**
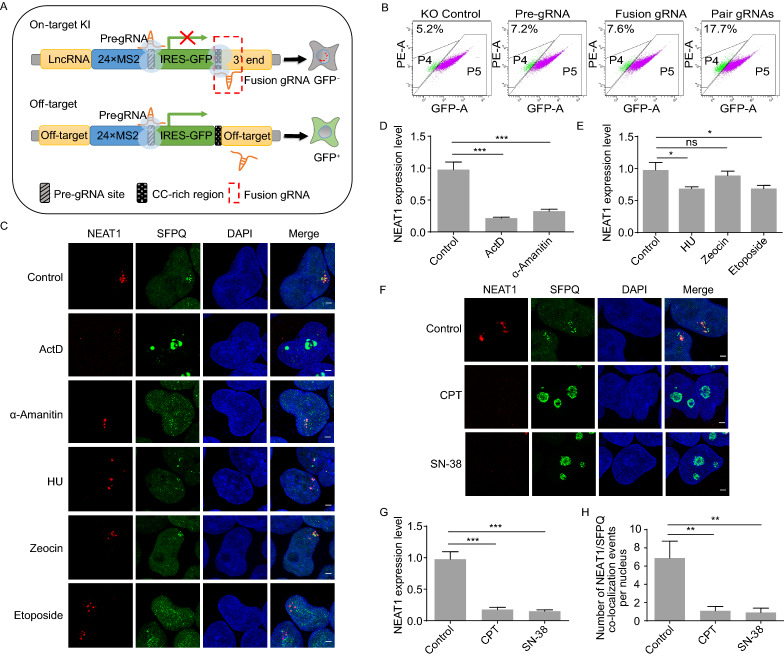
**Tracking of NEAT1 and paraspeckles dynamics under different stresses**. (A) Schematic of GFP negative sorting for on-target insertion cells after paired gRNAs KO. Paired gRNAs selected for on-target KI and would increase the proportion of GFP negative cells, while off-target insertions could not generate efficient GFP KO. (B) After paired gRNAs KO, the GFP negative cell population was enriched by FACs sorting. P2 was the gate for GFP negative selection. (C) Lentiviral expressed GFP-fused SFPQ in NEAT1 KI cells. NEAT and SFPQ merged well in the untreated KI cells. NEAT1 (red) and SFPQ (green) dynamics were recorded under different stimuli treatment over time. The concentration of ActD, α-Amanitin, HU, zeocin and etoposide was 250 ng/mL, 50 ng/mL, 2 μmol/L, 100 μg/mL and 100 μmol/L, respectively. Cells were fixed after 4 h treatment and nucleus were stained by DAPI (blue). (D) RT-qPCR analysis indicated that both ActD and α-Amanitin reduced the expression of NEAT1 significantly after 4 h treatment. (E) Quantification of NEAT1 expression level after different DNA damage stimuli treatment for 4 h by RT-qPCR. (F) Topoisomerase I inhibitors CPT (5 μmol/L) and SN-38 (1 μmol/L) diminished NEAT1 and relocated SFPQ to the nucleolus after 4 h treatment. (G) Both CPT and SN-38 treatment could dramatically reduce NEAT1 expression level, determined by RT-qPCR. Error bars represent SD in triplicate experiments. GAPDH was used as the internal control. (H) Co-localization of NEAT1/SFPQ foci per nucleus significantly reduced after CPT (5 μmol/L) and SN-38 (1 μmol/L) treatment. ns, no significant difference, *P < 0.01, **P < 0.001, ***P < 0.001 (Student’s *t* test)

To salvage the GFP marker for multi-color labeling as well as eliminate potential off-target insertions within cells, two gRNAs respectively targeting the pre-gRNA site and the CC-rich region-endogenous lncRNA junction were simultaneously introduced into the GFP positive cell lines with Cas9 (Figs. 1B and 4A). If the intact tagging cassette is precisely inserted into the lncRNA locus, further Cas9 cleavage should remove IRES-GFP and render the cells GFP-negative, while off-target insertions by error-prone NHEJ pathway or inefficient double knocked-out cells still expressing GFP. As expected, only the existence of paired gRNAs could significantly eliminate the GFP expression (Fig. 4B). After double KO by the paired gRNAs, the remained GFP positive cells were also subcloned and identified by genotyping PCR (Fig. S2). A majority of the GFP positive cells were still correctively inserted into the on-target loci as judged by PCR products with expected size, probably due to inefficient double KO of the IRES-GFP fragment simultaneously. The correctly tagged GFP negative cells were then enriched by a second round of GFP negative selection.

Proteins such as SFPQ and NONO are known to be key regulators of paraspeckles and interact with NEAT1 (Naganuma et al., 2012). To study the spatiotemporal distribution of NEAT1 in live cells, we stably expressed GFP-SFPQ fusion protein in the NEAT1-g1 KI cell line, and isolated cell clones with appropriate expression level that similar to endogenous SFPQ for further observation. Similarly, CERTIS-labeled NEAT1 co-localized well with GFP-SFPQ (Fig. 4C). Then different stimuli were added to the cells to track the NEAT1 and paraspeckles dynamics over time. With the addition of ActD, SFPQ aggregated into the nucleolus caps within a few hours, followed by rapid depolymerization of NEAT1 and disruption of paraspeckles. Interestingly, when the cells were treated with another RNA pol II inhibitor α-Amanitin, there was little SFPQ aggregated to the nucleolus caps while the paraspeckles were still stable, although the expression level of NEAT1 was also dramatically reduced after α-Amanitin treatment (Fig. 4C and 4D). This implies that there may be additional mechanism participates in the collapse of paraspeckles.

Since paraspeckles were previously speculated to be relative to DNA damage response, we further treated the cells with a series of DNA damage reagents to observe dynamic change of NEAT1 and paraspeckles. Hydroxyurea (HU) triggers fork stalling through inhibition of ribonucleotide reductase. We did not find response on paraspeckle distribution after HU treatment. The radiomimetic chemical zeocin could generate random DNA double-strand breaks (DSB) in the genome, while another DSB-trigger etoposide works by inhibiting topoisomerase II (Delacôte et al., 2007; Wu et al., 2011). Paraspeckles were insensitive to zeocin-induced DSB, with no significant morphological or distribution change even under prolonged treatment. Etoposide treatment for 4 h did not cause paraspeckle disruption, but we found reduced NEAT1 signals and some of SFPQ moved away from paraspeckles (Fig. 4C). RT-qPCR demonstrated that all of these drugs could lower NEAT1 expression level to some extent (Fig. 4E). Interestingly, when we treated the cells with DNA single-strand break (SSB) reagent CPT, the well-known topoisomerase I inhibitor, SFPQ accumulated into the nucleolus in a short time, resulting in NEAT1 diminishing and paraspeckle collapse. To further confirm the relationship between topoisomerase I inhibition and paraspeckle disruption, we treated the cells with SN-38, another topoisomerase I specific inhibitor. We noticed that SN-38 could generate the similar phenotype as CPT even at a very low concentration (1 μmol/L) (Fig. 4F). The corresponding expression level of NEAT1 were consistent with the phenotype (Fig. 4G), while the co-localization of NEAT1/SFPQ foci per nucleus was significantly reduced after CPT or SN-38 treatment, strongly indicating that paraspeckle may response to topoisomerase I defects. Besides, by using the NEAT1-labeled cells that before GFP excision, we found the regulatory relationship of CPT was also correctly mirrored by the GFP intensity through flow cytometer (Fig. S3A–C). These results indicated that paraspeckles may play multifunctional roles under different types of DNA damage. In addition, the CERTIS platform is suitable for *in vivo* tracking endogenous lncRNA dynamics, and for expedient screening of the lncRNA regulators.

### CERTIS enables long-term time-lapse imaging of endogenous NEAT1 in living cells

**Figure 5:**
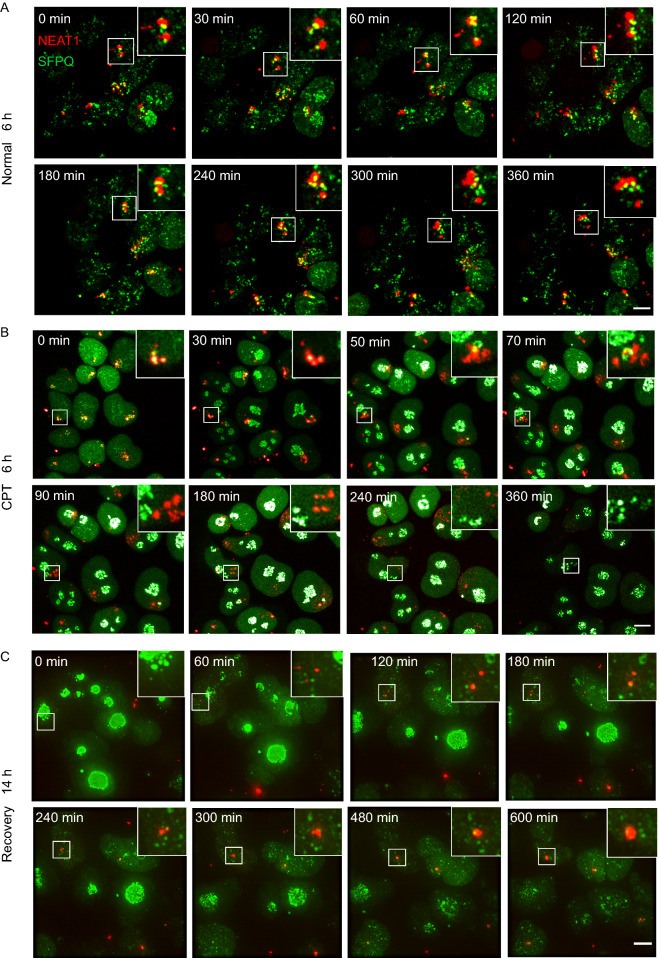
**CERTIS enables continuous, long-term live cell imaging of endogenous NEAT1 dynamics**. (A) Time-laps snapshots from live imaging of CERTIS-labeled NEAT1 (red). GFP-fused SFPQ (green) was stably expressed to monitor paraspeckle protein dynamic change. The movie was taken for 6 h with 15 min time-lapse. Insets showed the magnified boxed region of the merged channel of NEAT1 and its interaction with SFPQ. Scale bar: 2 μm. Also see Supplementary Movie S1. (B) 5 μmol/L CPT was added to the cells and the dynamic of NEAT1 and SFPQ were recorded with 15 min time-lapse for 6 h. Also see Supplementary Movie S2. (C) Snapshots from the live cell imaging of NEAT1 and SFPQ after removal of CPT medium. Paraspeckles recovery were recorded after CPT treatment for 6 h. The movie was taken for 14 h with 15 min time-lapse. Also see Supplementary Movie S4

To assess the long-term imaging performance of CERTIS and analyze paraspeckles dynamics in living cells, a time-lapse capture of CERTIS labeled NEAT1 was performed. GFP-SFPQ was also monitored as a reference for paraspeckle protein dynamics. First, the KI cells were cultured under normal condition, and images were taken in 3D z-stacks every 15 min for 6 h (Fig. 5A and Supplementary Movie S1). The mScarlet signal did not show photobleaching after 6 h observation. Notably, even in normal culture medium, the number and size of paraspeckles were dynamically altered, while SFPQ was consistently located inside or next to NEAT1 foci. Besides, a fraction of SFPQ scattered across the nucleus, suggesting the multi-functional character of SFPQ (Knott et al., 2016). Since we found that NEAT1 and paraspeckles were sensitive to topoisomerase I specific inhibitors, we next added CPT to the cells and recorded the NEAT1 and paraspeckles dynamics. We found SFPQ moving away from NEAT1 and relocating to the nucleolus rapidly. Consequently, NEAT1 broke up into smaller puncta within a few hours and finally dispersed into the nucleoplasm (Fig. 5B and Supplementary Movie S2). Similarly, when the RNA synthesis inhibitor ActD was added, a disruption of paraspeckles was successfully tracked while SFPQ depolymerized with NEAT1 (Fig. S4). To further evaluate CERTIS in long-term imaging and study reassemble of paraspeckles, we replaced the CPT culture medium with normal medium after 6 h treatment and performed live cell imaging for another 14 h. After removal of CPT, SFPQ gradually infiltrated from the nucleolus and reunited with NEAT1 puncta again, followed by regrouping of NEAT1 foci and finally formed bigger paraspeckles (Fig. 5C and Supplementary Movie S3). We suspected that this dynamic change of paraspeckles is similar to phase separation. Overall, these results demonstrated that CERTIS labeled method is suitable for long-term dynamic tracking of endogenous lncRNA. By virtue of CERTIS, we successfully recorded the reversible processes of the disruption and reconstitution of paraspeckles in live cells.

### Proteomic profiling of CERTIS labeled NEAT1 by native RIP-MS

**Figure 6:**
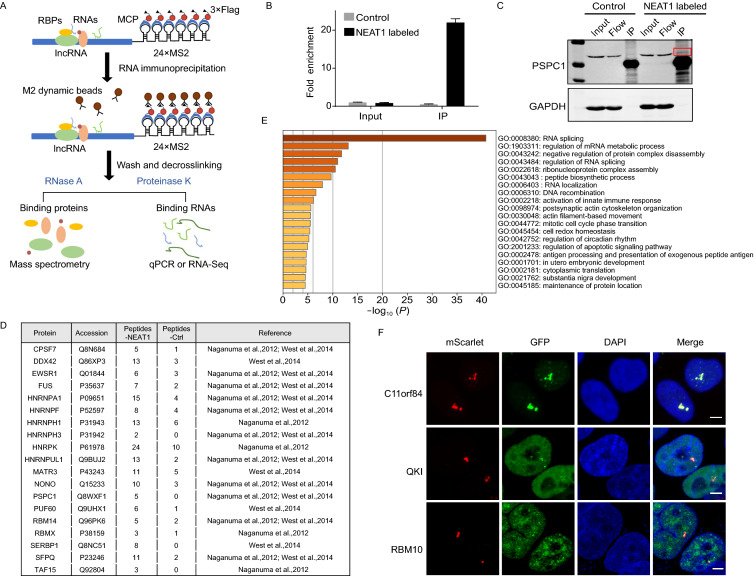
**Simple and efficient nRIP approach for isolation of lncRNA-protein complex of CERTIS labeled NEAT1**. (A) Overview of the workflow for nRIP of CERTIS labeled lncRNA. Cells are lysis by mild lysate and the labeled lncRNA is pulled-down by M2 dynamic beads through interaction between 3×Flag-tagged MCP and 24×MS2 sequence. The lncRNA-protein complex is digested by RNase A to release the binding proteins or proteinase K to release the interacting RNAs, followed by RIP-MS or RIP-qPCR/seq. (B) The efficiency of RNA immunoprecipitation was assayed by RIP from control or NEAT1 KI cells using M2 dynamic beads followed by RT-qPCR. Bar plots represent the fold enrichments of NEAT1 immunoprecipitated by M2 beads over the same amount of input across different samples, and error bars represent SD in triplicate experiments. U1snRNA was a control that does not bind to M2 beads. (C) Western blotting demonstrated that PSPC1 could be pulled-down by nRIP in NEAT1 KI cells. The band in red box indicates enriched PSPC1 protein after immunoprecipitation. (D) Cross-reference of the enriched proteins with previous identified paraspeckle component proteins. (E) GO Biological Process analysis revealed the top biological process category of enriched proteins from nRIP-MS result. GO analysis was performed using Metascape. (F) C11orf84, QKI and RBM10 were identified as new paraspeckle proteins that co-localized well with NEAT1. Candidate proteins were fused with a GFP tag and their co-localization with endogenous labeled NEAT1 (red) was determined. The nucleus was stained by DAPI (blue). Scale bar: 2 μm

To understand the function and regulatory mechanisms of lncRNAs, it is necessary to identify proteins that associate with lncRNAs. Current methods for identification of lncRNA-protein interactome mostly used RNA hybridization and are crosslinking-based, which is time-consuming and laborious (George et al., 2018). Since the MCP-fused protein contained epitope tags for immunoprecipitation, we developed an efficient and simple native RIP (nRIP) approach to identify proteins that interact with lncRNA. In our system, 3×Flag-fused MCP was applied to pull-down the MS2 tagging lncRNA because of the high affinity and specificity of Flag antibody. For CERTIS-based nRIP, the cells were lysis under a mild lysate, then the MCP-lncRNA-protein complexes were pulled down using M2 magnetic beads. After proteinase K treatment and RNA extraction using part of the samples, we could determinate the fold enrichment of the target lncRNA by q-PCR or for further usage such as RNA-seq. The rest of the samples were digested with RNase A to release the interacting proteins for western blot or mass spectrometry analysis (Fig. 6A). We found CERTIS-labeled NEAT1 was significant enriched after nRIP by q-PCR (Fig. 6B). Importantly, paraspeckle component PSPC1 was also enriched after immunoprecipitation when compared to the control (Fig. 6C). Next, NEAT1 associated proteins were identified by mass spectrometry. We cross-referenced our enrichment protein list with previous identified paraspeckle proteins according to two different genome-wide screening (Naganuma et al., 2012; West et al., 2014), and found many of the known paraspeckle-associated proteins were identified, suggesting CERTIS-based nRIP enables efficient pull-down of the lncRNA interacting proteins (Fig. 6D and Table S2). The overlapping proteins among three methods were also displayed and listed by the venn diagram (Fig. S5). Core paraspeckle component such as the Drosophila behavior/human splicing (DBHS) protein family and a series of RNA splicing factors were successfully identified. GO analysis showed that the enriched proteins were consistent with paraspeckle’s function as described before, mainly participating in the regulation of RNA splicing, protein complex disassembly, RNA localization, DNA recombination and innate immune response (Fig. 6E and Table S2). Notably, some of the proteins have not been reported to co-localize with NEAT1 or paraspeckles. To identify new paraspeckle proteins, we determined the localization of a few candidates using GFP fusions in NEAT1 KI cells. Among them, C11orf84, QKI and RBM10 colocalized well with NEAT1 puncta (Fig. 6F). The roles of these proteins in paraspeckles warrant further study in the near future. These results demonstrated that CERTIS-based nRIP could be a robust screening method for identifying lncRNA interacting proteins.

## Disscusion

Paraspeckles are a highly dynamical membraneless organelle that built on an architectural lncRNA NEAT1. Since the paraspeckle has been long discovered, yet its function and regulation have still been obscure. In this study, we successfully labeled 23 kb lncRNA NEAT1 using the newly developed CERTIS method. First, we demonstrated that knock-in of MS2-repeat does not affect the expression and physicochemical properties of endogenous NEAT1. Next, we showed the expression regulation of NEAT1 could be quantitatively monitored by detecting the GFP intensity through flow cytometry, paving ways for potential genome-wide screening of lncRNA regulators. Taking advantage of CERTIS, we performed short- and long-term tracking of NEAT1 dynamics under a series of drugs treatment, and successfully recorded the reversible process of NEAT1 disaggregation and reconstitution in living cells when treated or withdrew of topoisomerase I inhibitor CPT. At last, we developed an efficient approach for native RIP of the CERTIS labeled NEAT1, and successfully identified several new paraspeckle proteins.

We found that paraspeckles have different responses during different drugs treatment in this study. Interestingly, we observed that paraspeckle collapsed rapidly after the addition of topoisomerase I inhibitors CPT and SN-38, which could frequently generate single-strand DNA damage in the genome. It is also remarkable that although both ActD and α-Amanitin are transcription inhibitors, ActD, but not α-Amanitin could disrupt the formation of paraspeckle in a short time. The mechanism of them may be a little different, α-Amanitin is a specific inhibitor of RNA polymerases II and III (Weinmann et al., 1974), while ActD inhibits DNA-primed RNA synthesis by forming a stable complex with double-stranded DNA. Besides, ActD could cause single-strand breaks in DNA (Perry and Kelley, 1970; Roots and Smith, 1976; Sobell, 1985). According to the previous finding, we suspect that the disruption of paraspeckle after ActD treatment might also be caused by the frequent DNA SSB rather than transcription inhibition. In a word, the regulatory relationship between paraspeckle and DNA SSBR is worthy for further research in the future.

**Figure 7:**
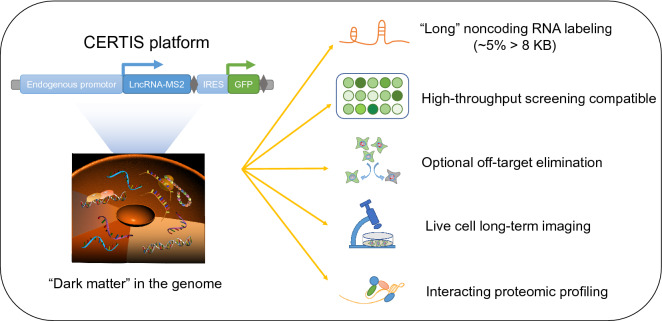
**Application and advantages of CERTIS platform in lncRNA biology**. The schematic diagram concludes the application and advantages of CERTIS platform in lncRNA biology. To study the dark matter in the genome, CERTIS allows precise and efficient knock-in of the MS2 cassette into the lncRNA. The second round knock-out for GFP negative selection is optional for eliminating the off-target insertions. CERTIS has an advantage on the labeling of very long LncRNA, which is hard to achieve through exogenous overexpress and tagging. The endogenous promoter-driven GFP enables quantitatively monitoring of the regulation of lncRNA in live cells, which is compatible for high-throughput gene inactivation or drug screening. By stably express the MCP-mScarlet-FLAG fusion protein, it allows for both short- and long-term tracking of the labeled lncRNA in live cells as well as studying the lncRNA-protein interactomes

In this work, we establish CERTIS as a promising tool for studying endogenous lncRNA-protein complexes, and the application and advantages of CERTIS platform were listed (Fig. 7). Precise insertion of MS2-repeat by CRISPR/Cas9-mediated knock-in enables tracking both expression level variation and spatiotemporal distribution of lncRNA, as well as screening for lncRNA-protein interactomes. After Cas9 cleavage, DNA double-strand breaks could be repaired accurately through several recombination pathways in the presence of different lengths of homologous arms, such as HR, HMEJ and MMEJ. Among them, MMEJ repair pathway needs only 20 nt micro-homology arms, which is much easier to prepare and is more efficient in certain cell lines than HR. The random off-target insertions by error-prone NHEJ pathway is a main concern of this field. Taking advantage of MMEJ short homology arms, the CC-rich region-endogenous lncRNA junction is predictable, which could form newly fusion gRNA sites at the C-terminal of insertion, while longer homologous arms usually used in HR template could not generate such a fusion. To eliminate off-target insertions, a pair of gRNAs respectively target the pre-gRNA site and CC-rich region-endogenous lncRNA junction could be further introduced to the GFP positive knocked-in cells and followed by another round of GFP negative sorting. Although there is still a possibility that off-target insertions could also harbor sequence similarity that targeted by our gRNAs, our method should have largely reduced the probability of random integration and tagging of irrelevant RNAs. Furthermore, other RNA labeling methods such as RNA FISH could help to eliminate the off-target events in the KI cells.

At present, there is still a lack in efficient methods for simultaneous tracking endogenous lncRNA dynamics and screening lncRNA interacting proteins in mammalian cells. Compared to the existing RNA labeling systems, CERTIS platform has an advantage on high-throughput screening of regulators that may affect lncRNA spatiotemporal dynamics in living cell, which are usually accomplished with the alteration of its interaction proteins. As a consequence, nRIP could be performed to identify differential interacting proteome under certain conditions, which may help us discover the new role of lncRNA plays in specific biological processes. The nRIP method also has some advantages over cross-linking method. Besides of easy accessibility, nRIP allows to reveal the identity of lncRNAs directly bound by the protein and their abundance in the immunoprecipitated samples. However, one drawback of nRIP is that the weak or indirect interaction protein of lncRNA might be omitted from the immunoprecipitation. Recently, a method called RaPID has been developed to detect the RNA-protein interaction based on proximity labeling, which allows to capture the weak and indirect interaction through biotinylation of the proximal proteins at high affinity (Ramanathan et al., 2018). When in combination with CERTIS by fusing BirA* with MCP, it is promising to identify the interactome of an endogenous lncRNA regardless of their interaction strength. However, like other proximity labeling BioID methods, RaPID can only identify protein in the proximity of the biotinylation enzyme. Thus, nRIP and RaPID would be important complementation for each other to unveil the interactive proteomics of the target lncRNA.

The superiority of the MS2-MCP system has long been confirmed in human cells, but mainly tested in overexpression conditions. However, large amounts of evidence have indicated that overexpression of RNAs may causes many false positive phenotypes and might not reveal the authentic regulation of endogenous RNAs. Moreover, exogenous overexpression and tagging of the very long lncRNAs (~5% over 8 kb in human genome) (Ma et al., 2014) in the cell is usually inefficient and hard to achieve. By combining CRISPR/Cas9-mediated KI with MS2-MCP system, we successfully developed an efficient system for both visualization and immunoprecipitation of endogenous lncRNA, such as NEAT1 23 kb in length. However, there are still some concerns about CERTIS labeling system. First, the insertion of MS2-repeat might affect the spatiotemporal distribution of certain lncRNAs. For the very long lncRNAs, this concern might be less. Second, for lncRNAs with three-dimensional structures at the 3′ end, it may be necessary to test suitable insertion site of lncRNA to prevent the misleading location or weakening MS2-MCP binding. To solve these problems, we expect that the MS2 repeats could be reduced to 12× or 6× according to the length of the target lncRNA, or other smaller RNA motif system such as boxB-λN could be alternatives for MS2-MCP (Kawakami et al., 2006).

In summary, as a novel lncRNA labeling system, CERTIS shows great potential on the deciphering of function and regulatory mechanisms of the highly dynamical lncRNAs such as NEAT1. Scaling-up the application of CERTIS on more lncRNAs is urgent to further optimized this system. Meanwhile, working along with other traditional methods can help us to obtain a more reliable and unbiased result of lncRNA biology. In the near future, we believe that CERTIS could become one of the universal tools in the field of lncRNA research and could be a help to flourish the study of lncRNAs.

## Materials and methods

### Plasmid construction and sgRNA preparation

To construct plenti-tdMCP-mScarlet-3×Flag, the NLS-tdMCP fragment was amplified from pHAGE-UBC-NLS-HA-tdMCP-GFP (a gift from Robert Singer; Addgene plasmid no.40649), and mScarlet-3×Flag fragment was amplified from the synthesized plasmid (IGE Biotechnology), then ligated together into a lentivirus vector containing an EF-1α promoter by NEBuilder HiFi DNA Assembly (NEB). The universal KI template plasmid was constructed by ligation of 24×MS2 and IRES-GFP fragment into the multiple cloning site of pBlueScript II SK(+) (Agilent) by NEBuilder HiFi DNA Assembly (NEB). 24×MS2 fragment was amplified from pHAGE-CMV-CFP-24×MS2 (a gift from Robert Singer; Addgene plasmid no.40651), and IRES-GFP fragment was amplified from pMSCV PIG (a gift from David Bartel; Addgene plasmid no.21654). The pre-gRNA site and CC-rich region was introduced into the fragments through overlapping PCR primers. The sequence of full-length universal KI template vector was shown in Fig. S6.

Full-length SFPQ and other paraspeckle candidate proteins were PCR amplified from the cDNA of HEK293T cells. The PCR products were first cloned into pENTR™ vector (Invitrogen) and then transferred into a Gateway-compatible destination vector containing a GFP tag by LR reaction according to the manufacturer’s protocol (Invitrogen).

The sgRNAs were designed on the website of https://chopchop.cbu.uib.no/# as described before (Montague et al., 2014). The sgRNA oligos were cloned into the pDR274 vector and were *in vitro* transcribed using the MEGAshortscript T7 transcription kit (Thermo Fisher). sgRNAs were purified using the MEGAclear kit (Thermo Fisher), then dissolved in RNase-free water and quantified using NanoDrop 1000 (Thermo Fisher). The target sequences of gRNAs were shown in Table S1.

### One-step generation of linear DNA donor

The universal KI template plasmid was used for generation of linear DNA donor as a PCR template. Primers (IGE Biotechnology) contain 20 nt micro-homology arms (MHAs) around the cleave site (3 nt upstream of PAM) and 25 to 30 nt overlapping region of the universal template plasmid. In addition, all primers were 5′ thiophosphorylated to enhance the half-life of linear donor fragments as previously reported (Orlando et al., 2010; Tasan et al., 2018) and further lengthen the phosphorothioate bonds to their last 5 nt of 5′ end. PCR was performed using KOD FX (TOYOBO) with a step-down cycle condition following manufacturer’s instructions. Then the PCR products were purified by Gel Extraction Kit (Qiagen) and dissolved in nuclease-free water. Sequence of the primers were shown in Table S1.

### MMEJ-based CRISPR KI

To generate highly efficient KI in the target locus,electroporation of Cas9 RNP and the linear DNA donor simultaneously to the cells was performed as previously reported (Kim et al., 2014). Specifically, 10 μg TrueCut Cas9 Protein v2 (Thermo) and 2 μg *in vitro* transcribed sgRNA were incubated at 37 °C for 20 min, and 5 × 10^5^ HEK293T cells (70%–90% confluent) were harvested and centrifuged at 90 ×*g* for 10 min. Then the Cas9 RNP was electroporated to the cell pellet together with 1 μg linear DNA donor by Lonza 4D-Nucleofector (Lonza) under a pre-optimized program. 72 h after electroporation, GFP positive cells were enriched by FACs sorting (BD FACSAria II) as described before (Lee et al., 2011), followed by another two rounds of consecutive sorting to make sure the cell population was nearly 100% GFP positive. Then the KI cells were visualized by the stably expression of tdMCP-mScarlet-3×Flag fusion protein. Single cell clones were isolated for further genotyping and experiments. For the precise excision of IRES-GFP fragment, a pair of gRNAs that respectively targeted the pre-gRNA site and the GFP-NEAT1 fusion junction were *in vitro* transcribed and simultaneously electroporated to the GFP positive clones with Cas9 protein. 72 h after electroporation, GFP negative cells were enriched by another new round of FACs sorting.

### Cell culture, RNAi, lentiviral production and stable cell line construction

HEK293T cells were cultured in DMEM media supplemented with 10% fetal bovine serum and 1% penicillin-streptomycin. siRNA oligos of SFPQ and the negative control (siNC) were ordered from RiboBio and transfected into cells using Lipofectamine® RNAiMAX (Thermo Fisher Scientific). The sequence of oligos were the same as previously described (Naganuma et al., 2012) and were listed in Table S1.

Lentiviral particles were produced by transient transfection of HEK293T cells with pLenti-tdMCP-mScarlet-3×Flag, psPAX2, and pMD2.G in a ratio of 4:3:1. The supernatant containing viral particles was harvested twice at 48 h and 72 h after transfection, and virus titer was tested by TransLvTM Lentivirus qPCR Titration Kit (Transgene Biotech) following the manufacturer’s instructions. To construct a stable cell line with low expression level of tdMCP cassette, the MOI (Multiplicity of Infection) was controlled under 0.3 and cells were cultured in medium containing lentivirus and 8 μg/mL polybrene (Sigma) for 48 h, and then subjected to puromycin (5 μg/mL) selection for another 72 h. A low virus titer of lentiviral infection is critical to increase the SNR of the labeled lncRNA within the nucleus.

### Clonal isolation, genotyping and sequencing

Clonal isolation was started by limiting dilution in 96-well plate as described before (Mali et al., 2013). Once the clones grown in 12-well plates reached ~80% confluency, half of the cells were collected for genomic DNA (gDNA) extraction (Qiagen) and the other cells were preserved in liquid nitrogen until further use. Genotyping and Sanger DNA sequencing were carried out as described previously (Tasan et al., 2018). Briefly, all the genotyping PCRs were performed using KOD FX polymerase (TOYOBO) according to the manufacturer’s protocol. For the clones with expected genotyping results, knock-in band was purified by Gel Extraction Kit (Qiagen) and performed Sanger DNA sequencing (IGE biotech) to verify sequences of the 5′ and 3′ junctions as well as the integrity of the MS2 repeats. Sequences of the junction PCR primers can be found in Table S1.

### RNA isolation and qRT-PCR

Total RNA from each cultured cell line or cultured cells with different treatments was extracted by Trizol Reagent (Invitrogen). For the exact quantification of NEAT1 expression, an improved RNA extraction method specific to nuclear body-associated architectural lncRNA was used by needle shearing of cell lysate in the Trizol reagent as previously described (Chujo et al., 2017). A total of 1 μg RNA was treated with the RNase-free RQ DNase I (Promega) for 10 min at 37 °C and then reverse transcribed using the SuperScript III (Invitrogen) with oligo (dT) and random hexamers. Quantitative polymerase chain reaction (qPCR) was carried out in Applied Biosystems StepOne™ Real-Time PCR Systems using the GoTaq qPCR Master Mix (Promega). The relative expression of different sets of genes was quantified to GAPDH mRNA. Primer sequences for qRT-PCR were listed in Table S1.

### RNA FISH and immunofluorescence

HEK293T cells were grown on a glass slide and fixed in 4% formaldehyde in PBS. Cells were washed once with PBS, permeabilized with Triton X-100, dehydrated through a series of ethanol washes and hybridized overnight with a digoxigenin-labeled NEAT1 probe (Table S1). RNA FISH signal was detected by incubating with Alexa Flour 647-labeled anti-digoxygenin antibody (Roche) and examined on a Leica TCS SP8 STED microscope.

Immunofluorescence was carried out as described previously (Deng et al., 2019). Briefly, cells growing on glass slides were rinsed with PBS and fixed with 4% PFA for 15 min, and then permeabilized in 0.2% Triton X-100 followed by 30 min 5% BSA blocking. The cells were then stained with human anti-PSPC1 (Abcam, ab104238) or anti-SFPQ antibodies (Abcam, ab11825) in blocking buffer for overnight at 4 °C and washed three times with PBS. Then stained with Alexa Flour 647-labeled secondary antibodies (Jackson Laboratory) in blocking buffer for 1 h. After three washes with PBS, coverslips were mounted with VECTASHIELD mounting medium containing 0.5 μg/mL DAPI and then visualized at 100× on a CCD camera mounted on a Leica TCS SP8 STED microscope using imaging software. The magenta signal was defined as green by ImageJ (National Institutes of Health) before further colocalization analysis.

### Image acquisition and analysis

For fixed cells on a slide with a coverslip, images were acquired on a Leica TCS SP8 STED microscope using the confocal mode with a 100× oil immersion/1.4 N.A. objective (Leica Microsystems) following manufacturer’s instructions. For long-term imaging of living cells, a 93× glycerol immersion/1.4 N.A. objective was used and the cells were cultured in live cell workstation under 37 °C with 5% CO_2_. The excitation wavelengths were used according to the dye of the fluorescent protein specification: DAPI with 405 nm, GFP with 488 nm, mScarlet with 561 nm and Alexa Flour 647 with 633 nm. Emission was detected with a PMT detector. Gain and off-set were set at values which prevented saturated and empty pixels. Images of 1,024 × 1,024 pixels were obtained. 2D STED images as single planes or z-sectioning step with a slice distance of 300 nm were recorded and regions of interest were adjusted manually. After image acquisition, images were deconvolved under lightning model using Leica LAS Lite Software (Leica). Three-dimensional projection of the acquired movies was generated using maximum intensity in ImageJ. Image analyses of signal intensity and colocalization were also carried out by ImageJ software as reported before (Shao et al., 2016).

### Native RNA immunoprecipitation

Native RIP was carried out according to previously described (Xing et al., 2017). NEAT1-labeled cells (2 × 10^7^) were harvested and rinsed twice with ice-cold PBS, WT HEK293T stably expressing tdMCP-mScarlet-3×Flag was used as a control. Cells were suspended in 1 mL RNA immunoprecipitation (RIP) buffer containing 50 mmol/L Tris pH 7.4, 150 mmol/L NaCl, 0.5% NP-40, 1× RNasin plus (Promega), 2 mmol/L ribonucleoside vanadyl complex (VRC, NEB), 1 mmol/L PMSF and 1× protease inhibitor cocktail (Sigma). Followed by brief sonication, cell lysates were centrifuged at 10,000 ×*g* for 10 min at 4 °C and the supernatants were precleared with 10 μL Dynabeads Protein G (Invitrogen). The precleared supernatants were then divided into two parts equally and incubated with 20 μL anti-FLAG M2 Magnetic Beads (Sigma) for 2 h at 4 °C, followed by washing three times with RIP buffer. One-third of the beads were incubated with elution buffer (100 mmol/L Tris pH 6.8, 4% SDS, 10 mmol/L EDTA) at room temperature for 10 min followed by western blotting. The remainder was decrosslinking by proteinase K buffer (50 mmol/L Tris pH 7.4, 150 mmol/L NaCl, 1× RNasin plus, 0.5% SDS and 200 μg/mL proteinase K) at room temperature for 30 min before used for RNA extraction. For mass spectrometry, the binding proteins were released from the beads by incubating with the RNase A buffer (50 mmol/L Tris pH 7.4, 150 mmol/L NaCl and 1 μg/mL RNase A) before trypsin digestion. Liquid chromatography-tandem MS (LC-MS/MS) was carried out on the Thermo Q Exactive Hybrid quadrupole orbitrap mass spectrometer as reported before (Tsai et al., 2011).

## Electronic supplementary material

Below is the link to the electronic supplementary material.

Supplementary material 1 (PDF 515 kb)

Supplementary material 2 (MOV 2191 kb)

Supplementary material 3 (MOV 2966 kb)

Supplementary material 4 (MOV 7426 kb)

Supplementary material 5 (XLSX 12 kb)

Supplementary material 6 (XLSX 166 kb)

Supplementary material 7 (DOCX 1998 kb)

## References

[CR1] Bae S, Kweon J, Kim HS, Kim J (2014). Microhomology-based choice of Cas9 nuclease target sites. Nat Methods.

[CR2] Bindels DS, Haarbosch L, van Weeren L, Postma M, Wiese KE, Mastop M, Aumonier S, Gotthard G, Royant A, Hink MA (2017). mScarlet: a bright monomeric red fluorescent protein for cellular imaging. Nat Methods.

[CR3] Cao M, Zhao J, Hu G (2019). Genome-wide methods for investigating long noncoding RNAs. Biomed Pharmacother.

[CR4] Chen L (2016). Linking long noncoding RNA localization and function. Trends Biochem Sci.

[CR5] Chen L, Carmichael GG (2009). Altered nuclear retention of mRNAs containing inverted repeats in human embryonic stem cells: functional role of a nuclear noncoding RNA. Mol Cell.

[CR6] Chujo T, Yamazaki T, Kawaguchi T, Kurosaka S, Takumi T, Nakagawa S, Hirose T (2017). Unusual semi-extractability as a hallmark of nuclear body-associated architectural noncoding RNAs. EMBO J.

[CR7] Clemson CM, Hutchinson JN, Sara SA, Ensminger AW, Fox AH, Chess A, Lawrence JB (2009). An architectural role for a nuclear noncoding RNA: NEAT1 RNA is essential for the structure of paraspeckles. Mol Cell.

[CR8] Cox DBT, Gootenberg JS, Abudayyeh OO, Franklin B, Kellner MJ, Joung J, Zhang F (2017). RNA editing with CRISPR-Cas13. Science (New York, N.Y.).

[CR9] Delacôte F, Deriano L, Lambert S, Bertrand P, Saintigny Y, Lopez BS (2007). Chronic exposure to sublethal doses of radiation mimetic Zeocin™ selects for clones deficient in homologous recombination. Mutat Res.

[CR10] Deng T, Huang Y, Weng K, Lin S, Li Y, Shi G, Chen Y, Huang J, Liu D, Ma W (2019). TOE1 acts as a 3′ exonuclease for telomerase RNA and regulates telomere maintenance. Nucleic Acids Res.

[CR11] Derrien T, Guigó R, Johnson R (2012). The long non-coding RNAs: a new (P)layer in the “Dark Matter”. Front Genet.

[CR12] Evans JR, Feng FY, Chinnaiyan AM (2016). The bright side of dark matter: lncRNAs in cancer. J Clin Invest.

[CR13] Fox AH, Bond CS, Lamond AI (2005). P54nrb forms a heterodimer with PSP1 that localizes to paraspeckles in an RNA-dependent manner. Mol Biol Cell.

[CR14] Fox AH, Lam YW, Leung AK, Lyon CE, Andersen J, Mann M, Lamond AI (2002). Paraspeckles: a novel nuclear domain. Curr Biol.

[CR16] Fox AH, Nakagawa S, Hirose T, Bond CS (2018). Paraspeckles: where long noncoding RNA meets phase separation. Trends Biochem Sci.

[CR17] Fujimoto A, Furuta M, Totoki Y, Tsunoda T, Kato M, Shiraishi Y, Tanaka H, Taniguchi H, Kawakami Y, Ueno M (2016). Whole-genome mutational landscape and characterization of noncoding and structural mutations in liver cancer. Nat Genet.

[CR18] Fusco D, Accornero N, Lavoie B, Shenoy SM, Blanchard J, Singer RH, Bertrand E (2003). Single mRNA molecules demonstrate probabilistic movement in living mammalian cells. CURR Biol.

[CR19] George L, Indig FE, Abdelmohsen K, Gorospe M (2018). Intracellular RNA-tracking methods. Open Biol.

[CR20] Hirose T, Virnicchi G, Tanigawa A, Naganuma T, Li R, Kimura H, Yokoi T, Nakagawa S, Benard M, Fox AH (2014). NEAT1 long noncoding RNA regulates transcription via protein sequestration within subnuclear bodies. Mol Biol Cell.

[CR21] Hu S, Yao R, Chen L (2016). Shedding light on paraspeckle structure by super-resolution microscopy. J Cell Biol.

[CR22] Hutchinson JN, Ensminger AW, Clemson CM, Lynch CR, Lawrence JB, Chess A (2007). A screen for nuclear transcripts identifies two linked noncoding RNAs associated with SC35 splicing domains. BMC Genomics.

[CR23] Imamura K, Imamachi N, Akizuki G, Kumakura M, Kawaguchi A, Nagata K, Kato A, Kawaguchi Y, Sato H, Yoneda M (2014). Long noncoding RNA NEAT1-dependent SFPQ relocation from promoter region to paraspeckle mediates IL8 expression upon immune stimuli. Mol Cell.

[CR24] Itzkovitz S, van Oudenaarden A (2011). Validating transcripts with probes and imaging technology. Nat Methods.

[CR25] Jandura A, Krause HM (2017). The new RNA world: growing evidence for long noncoding RNA functionality. Trends Genet.

[CR26] Kawakami J, Sugimoto N, Tokitoh H, Tanabe Y (2006). A novel stable RNA pentaloop that interacts specifically with a motif peptide of lambda-N protein. Nucleosides Nucleotides Nucleic Acids.

[CR27] Kim S, Kim D, Cho SW, Kim J, Kim JS (2014). Highly efficient RNA-guided genome editing in human cells via delivery of purified Cas9 ribonucleoproteins. Genome Res.

[CR28] Kim SH, Vieira M, Kim H, Kesawat MS, Park HY (2019). MS2 labeling of endogenous beta-actin mRNA does not result in stabilization of degradation intermediates. Mol Cells.

[CR29] Knott GJ, Bond CS, Fox AH (2016). The DBHS proteins SFPQ, NONO and PSPC1: a multipurpose molecular scaffold. Nucleic Acids Res.

[CR30] Kopp F, Mendell JT (2018). Functional classification and experimental dissection of long noncoding RNAs. Cell.

[CR31] Kostyrko K, Mermod N (2016). Assays for DNA double-strand break repair by microhomology-based end-joining repair mechanisms. Nucleic Acids Res.

[CR32] Lanzós A, Carlevaro-Fita J, Mularoni L, Reverter F, Palumbo E, Guigó R, Johnson R (2017). Discovery of cancer driver long noncoding RNAs across 1112 tumour genomes: new candidates and distinguishing features. Sci Rep UK.

[CR33] Lee M, Sadowska A, Bekere I, Ho D, Gully BS, Lu Y, Iyer KS, Trewhella J, Fox AH, Bond CS (2015). The structure of human SFPQ reveals a coiled-coil mediated polymer essential for functional aggregation in gene regulation. Nucleic Acids Res.

[CR34] Lee O, Kim H, He Q, Baek HJ, Yang D, Chen L, Liang J, Chae HK, Safari A, Liu D (2011). Genome-wide YFP fluorescence complementation screen identifies new regulators for telomere signaling in human cells. Mol Cell Proteomics.

[CR35] Levsky JM, Singer RH (2003). Fluorescence in situ hybridization: past, present and future. J CELL SCI.

[CR36] Lionnet T, Czaplinski K, Darzacq X, Shav-Tal Y, Wells AL, Chao JA, Park HY, de Turris V, Lopez-Jones M, Singer RH (2011). A transgenic mouse for in vivo detection of endogenous labeled mRNA. Nat Methods.

[CR37] Liu S, Zhu J, Jiang T, Zhong Y, Tie Y, Wu Y, Zheng X, Jin Y, Fu H (2015). Identification of lncRNA MEG3 binding protein using MS2-tagged RNA affinity purification and mass spectrometry. Appl Biochem Biotech.

[CR38] Ma L, Bajic VB, Zhang Z (2014). On the classification of long non-coding RNAs. RNA Biol.

[CR39] Mali P, Yang L, Esvelt KM, Aach J, Guell M, DiCarlo JE, Norville JE, Church GM (2013). RNA-guided human genome engineering via Cas9. Science.

[CR40] Marchese FP, Raimondi I, Huarte M (2017). The multidimensional mechanisms of long noncoding RNA function. Genome Biol.

[CR41] Montague TG, Cruz JM, Gagnon JA, Church GM, Valen E (2014). CHOPCHOP: a CRISPR/Cas9 and TALEN web tool for genome editing. Nucleic Acids Res.

[CR42] Munschauer M, Nguyen CT, Sirokman K, Hartigan CR, Hogstrom L, Engreitz JM, Ulirsch JC, Fulco CP, Subramanian V, Chen J (2018). The NORAD lncRNA assembles a topoisomerase complex critical for genome stability. Nature.

[CR43] Naganuma T, Nakagawa S, Tanigawa A, Sasaki YF, Goshima N, Hirose T (2012). Alternative 3′-end processing of long noncoding RNA initiates construction of nuclear paraspeckles. EMBO J.

[CR44] Nakade S, Tsubota T, Sakane Y, Kume S, Sakamoto N, Obara M, Daimon T, Sezutsu H, Yamamoto T, Sakuma T (2014). Microhomology-mediated end-joining-dependent integration of donor DNA in cells and animals using TALENs and CRISPR/Cas9. Nat Commun.

[CR45] Nakagawa S, Shimada M, Yanaka K, Mito M, Arai T, Takahashi E, Fujita Y, Fujimori T, Standaert L, Marine JC (2014). The lncRNA Neat1 is required for corpus luteum formation and the establishment of pregnancy in a subpopulation of mice. Development.

[CR46] Nelles DA, Fang MY, O Connell MR, Xu JL, Markmiller SJ, Doudna JA, Yeo GW (2016). Programmable RNA tracking in live cells with CRISPR/Cas9. Cell.

[CR47] Nguyen VT, Kiss T, Michels AA, Bensaude O (2001). 7SK small nuclear RNA binds to and inhibits the activity of CDK9/cyclin T complexes. Nature.

[CR48] Orlando SJ, Santiago Y, DeKelver RC, Freyvert Y, Boydston EA, Moehle EA, Choi VM, Gopalan SM, Lou JF, Li J (2010). Zinc-finger nuclease-driven targeted integration into mammalian genomes using donors with limited chromosomal homology. Nucleic Acids Res.

[CR49] Park HY, Lim H, Yoon YJ, Follenzi A, Nwokafor C, Lopez-Jones M, Meng X, Singer RH (2014). Visualization of dynamics of single endogenous mRNA labeled in live mouse. Science.

[CR50] Perry RP, Kelley DE (1970). Inhibition of RNA synthesis by actinomycin D: characteristic dose-response of different RNA species. J Cell Physiol.

[CR51] Qin P, Parlak M, Kuscu C, Bandaria J, Mir M, Szlachta K, Singh R, Darzacq X, Yildiz A, Adli M (2017). Live cell imaging of low- and non-repetitive chromosome loci using CRISPR-Cas9. Nat Commun.

[CR52] Ramanathan M, Majzoub K, Rao DS, Neela PH, Zarnegar BJ, Mondal S, Roth JG, Gai H, Kovalski JR, Siprashvili Z (2018). RNA–protein interaction detection in living cells. Nat Methods.

[CR53] Rau K, Rentmeister A (2016). CRISPR/Cas9: a new tool for RNA imaging in live cells. ChemBioChem.

[CR54] Roots R, Smith KC (1976). Effects of actinomycin D on cell cycle kinetics and the DNA of Chinese hamster and mouse mammary tumor cells cultivated in vitro. Cancer Res.

[CR55] Sasaki YTF, Ideue T, Sano M, Mituyama T, Hirose T (2009). MEN[epsilon]/[beta] noncoding RNAs are essential for structural integrity of nuclear paraspeckles. Proc Natl Acad Sci USA.

[CR56] Shao S, Zhang W, Hu H, Xue B, Qin J, Sun C, Sun Y, Wei W, Sun Y (2016). Long-term dual-color tracking of genomic loci by modified sgRNAs of the CRISPR/Cas9 system. Nucleic Acids Res.

[CR57] Shelkovnikova TA, Robinson HK, Troakes C, Ninkina N, Buchman VL (2014). Compromised paraspeckle formation as a pathogenic factor in FUSopathies. Hum Mol Genet.

[CR58] Sobell HM (1985). Actinomycin and DNA Transcription. Proc Natl Acad Sci USA.

[CR59] Spille JH, Hecht M, Grube V, Cho WK, Lee C, Cisse II (2019). A CRISPR/Cas9 platform for MS2-labelling of single mRNA in live stem cells. Methods.

[CR60] Standaert L, Adriaens C, Radaelli E, Van Keymeulen A, Blanpain C, Hirose T, Nakagawa S, Marine J (2014). The long noncoding RNA Neat1 is required for mammary gland development and lactation. RNA.

[CR61] Sunwoo H, Dinger ME, Wilusz JE, Amaral PP, Mattick JS, Spector DL (2008). MEN/nuclear-retained non-coding RNAs are up-regulated upon muscle differentiation and are essential components of paraspeckles. Genome Res.

[CR62] Taleei R, Nikjoo H (2013). Biochemical DSB-repair model for mammalian cells in G1 and early S phases of the cell cycle. Mutat Res.

[CR63] Tasan I, Sustackova G, Zhang L, Kim J, Sivaguru M, HamediRad M, Wang Y, Genova J, Ma J, Belmont AS (2018). CRISPR/Cas9-mediated knock-in of an optimized TetO repeat for live cell imaging of endogenous loci. Nucleic Acids Res.

[CR64] Tsai BP, Wang X, Huang L, Waterman ML (2011). Quantitative profiling of in vivo-assembled RNA-protein complexes using a novel integrated proteomic approach. Mol Cell Proteomics.

[CR65] Tutucci E, Vera M, Biswas J, Garcia J, Parker R, Singer RH (2018). An improved MS2 system for accurate reporting of the mRNA life cycle. Nat Methods.

[CR66] Tutucci E, Vera M, Singer RH (2018). Single-mRNA detection in living S. cerevisiae using a re-engineered MS2 system. Nat Protoc.

[CR67] Wang Y, Hu S, Wang M, Yao R, Wu D, Yang L, Chen L (2018). Genome-wide screening of NEAT1 regulators reveals cross-regulation between paraspeckles and mitochondria. Nat Cell Biol.

[CR68] Wang Z, Fan P, Zhao Y, Zhang S, Lu J, Xie W, Jiang Y, Lei F, Xu N, Zhang Y (2017). NEAT1 modulates herpes simplex virus-1 replication by regulating viral gene transcription. Cell Mol Life Sci.

[CR69] Weinmann R, Raskas HJ, Roeder RG (1974). Role of DNA-dependent RNA polymerases II and III in transcription of the adenovirus genome late in productive infection. Proc Natl Acad Sci USA.

[CR70] Weinrich SL, Pruzan R, Ma L, Ouellette M, Tesmer VM, Holt SE, Bodnar AG, Lichtsteiner S, Kim NW, Trager JB (1997). Reconstitution of human telomerase with the template RNA component hTR and the catalytic protein subunit hTRT. Nat Genet.

[CR71] West JA, Davis CP, Sunwoo H, Simon MD, Sadreyev RI, Wang PI, Tolstorukov MY, Kingston RE (2014). The long noncoding RNAs NEAT1 and MALAT1 bind active chromatin sites. Mol Cell.

[CR72] West JA, Mito M, Kurosaka S, Takumi T, Tanegashima C, Chujo T, Yanaka K, Kingston RE, Hirose T, Bond C (2016). Structural, super-resolution microscopy analysis of paraspeckle nuclear body organization. J Cell Biol.

[CR73] Wu B, Chao JA, Singer RH (2012). Fluorescence fluctuation spectroscopy enables quantitative imaging of single mRNAs in living cells. Biophys J.

[CR74] Wu C, Li T, Farh L, Lin L, Lin T, Yu Y, Yen T, Chiang C, Chan N (2011). Structural basis of type II topoisomerase inhibition by the anticancer drug etoposide. Science.

[CR75] Xing Y, Yao R, Zhang Y, Guo C, Jiang S, Xu G, Dong R, Yang L, Chen L (2017). SLERT regulates DDX21 Rings associated with Pol I transcription. Cell.

[CR76] Yang LZ, Wang Y, Li SQ, Yao RW, Luan PF, Wu H, Carmichael GG, Chen LL (2019). Dynamic imaging of RNA in living cells by CRISPR-Cas13 systems. Mol Cell.

[CR77] Yang Y, Wen L, Zhu H (2015). Unveiling the hidden function of long non-coding RNA by identifying its major partner-protein. Cell Biosci.

[CR78] Yao R, Wang Y, Chen L (2019). Cellular functions of long noncoding RNAs. Nat Cell Biol.

[CR79] Zhang Q, Chen CY, Yedavalli VS, Jeang KT (2013). NEAT1 long noncoding RNA and paraspeckle bodies modulate HIV-1 posttranscriptional expression. MBIO.

