# Supplementary material for: Live cell imaging and proteomic profiling of endogenous NEAT1 lncRNA by CRISPR/Cas9-mediated knock-in
Source: Protein Cell. 2020 May 26;11(9):641–60. doi: 10.1007/s13238-020-00706-w (PMC7452982; doi:10.1007/s13238-020-00706-w)
Supplement: Supplementary file 1 — Supplementary material 1 (PDF 515 kb) [file 13238_2020_706_MOESM1_ESM.pdf]

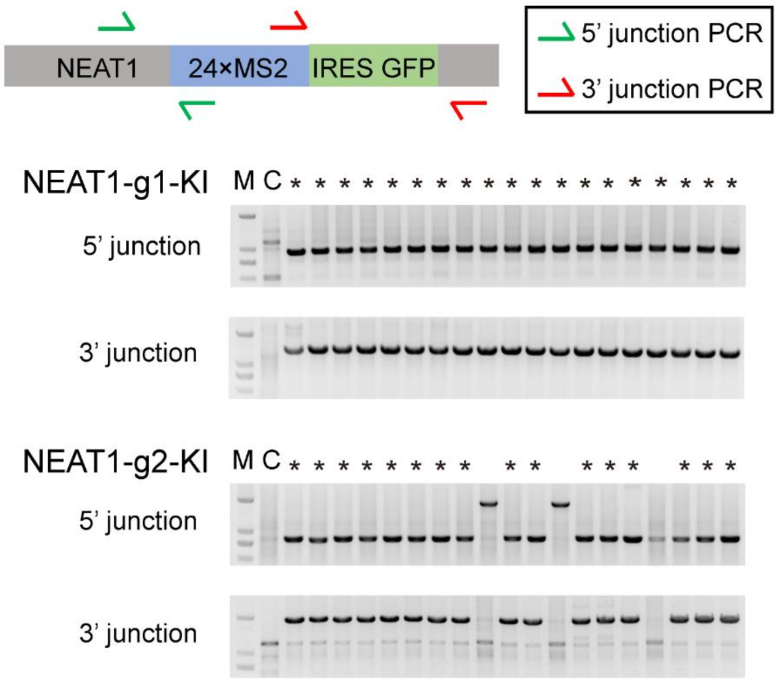

Supplementary Figure S1. Junction PCR of the single clones from two independent KI cell lines. Upper panel shows the schematic of the 5’ and 3’ junction PCR primers used for genotyping. Lower panel shows the junction PCR of the single clones of two KI cell lines. Expected band sizes from the knock-in alleles of line1 are around 0.9 KB for 5’ junction PCR and 1.4 KB for 3’ junction PCR, while line2 are around 0.6 KB for 5’ junction PCR and 1.9 KB for 3’ junction PCR. WT HEK293T gDNA was used as a negative control. C indicates WT control, * indicate single clones with expected band size.

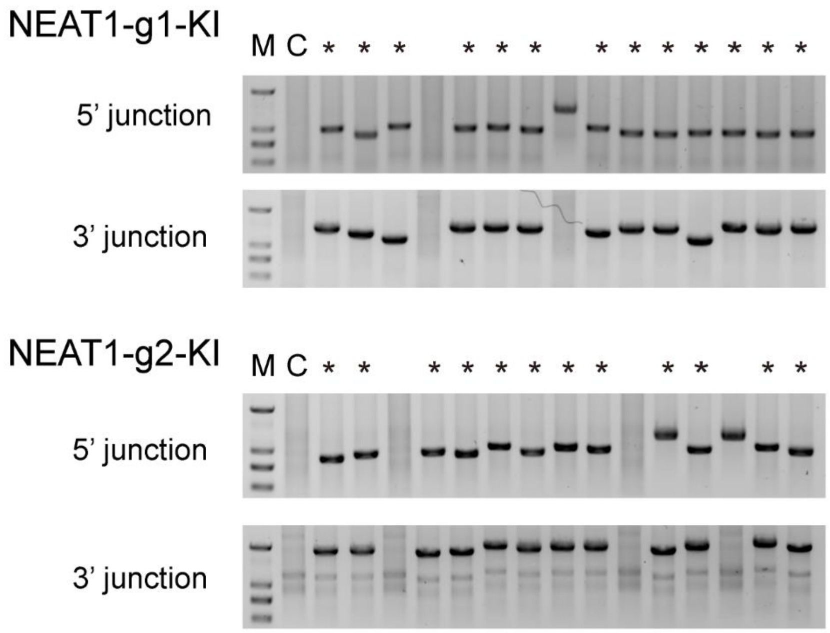

Supplementary Figure S2. Genotyping PCR of the GFP positive subclones from two NEAT1 KI cell lines after paired gRNAs KO. 5’ and 3’ junction PCR were carried out for the GFP positive subclones of two NEAT1 KI cell lines after double KO of IRES-GFP fragment. On-target KI cells without simultaneous cutting of paired gRNAs would still retain the expected band sizes of PCR products and GFP expression, while off-target insertions could not generate the expected junction PCR products. WT HEK293T gDNA was used as a negative control. C indicates WT control, * indicate single clones with expected band size.

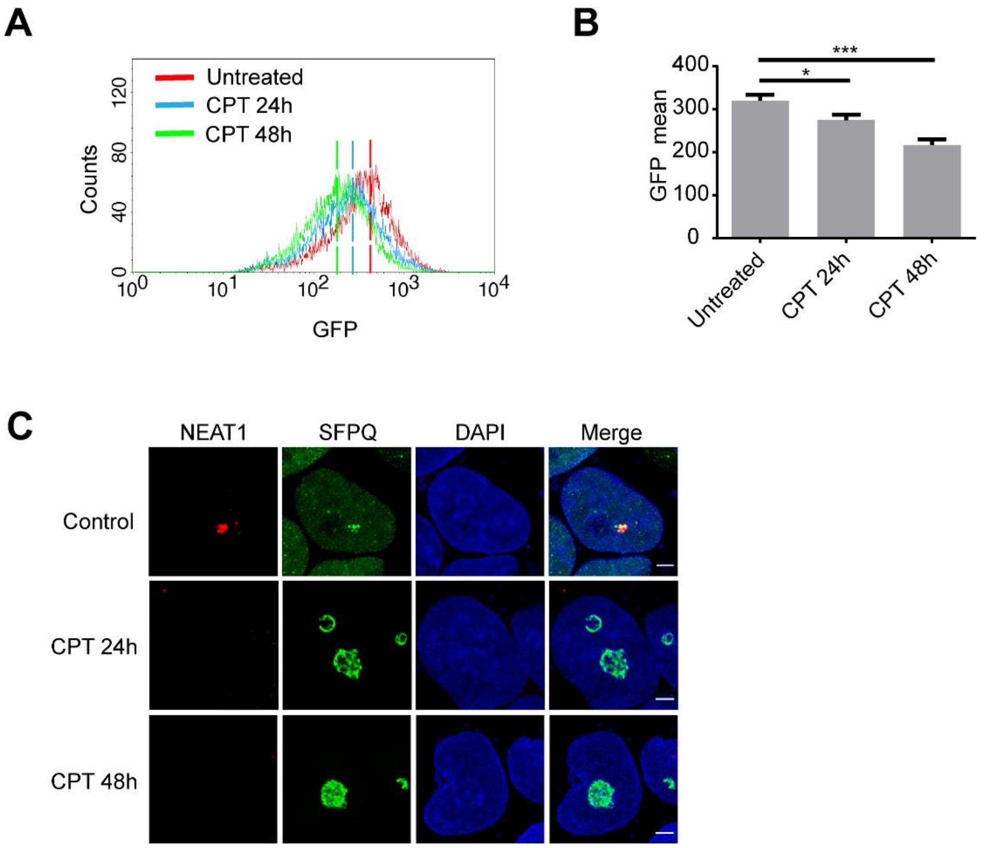

Supplementary Figure S3. GFP intensity of CERTIS labeled NEAT1 correctly mirror the regulatory relationship by CPT in live cells

(A) GFP peak shifted to the left gradually after 5 μM CPT treatment for 24 and 48 h, respectively. Untreated cells were used as control. GFP fluorescence intensity was detected by flow cytometer and the dotted lines indicated the mean of GFP fluorescence intensity.

(B) Mean fluorescence intensity (from A) showed time-dependent decrease of NEAT1 level after CPT treatment for 24 and 48 h.

(C) NEAT1 (red) disaggregated and SFPQ relocated to the nucleolus after CPT treatment. Scale bar: 2 μm.

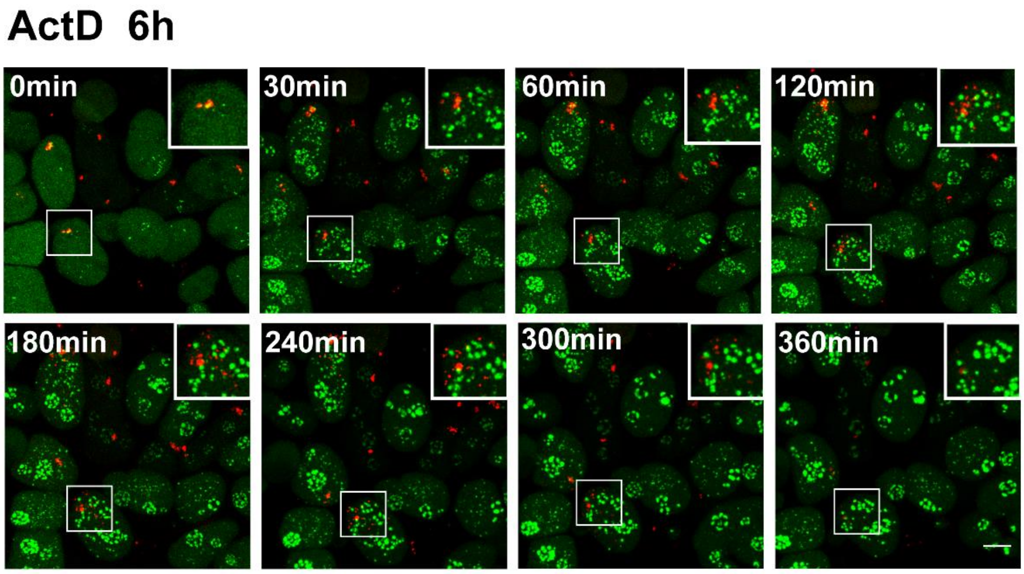

Supplementary Figure S4. CERTIS enables long-term time-lapse imaging of endogenous NEAT1 in living cells. Snapshots displayed the dynamic of NEAT1 and SFPQ after 250 ng/ml ActD treatment for 6 h, with a 15 min time-lapse. Insets showed the magnified boxed region of the merged channel of NEAT1 and SFPQ. Scale bar: 2 μm.

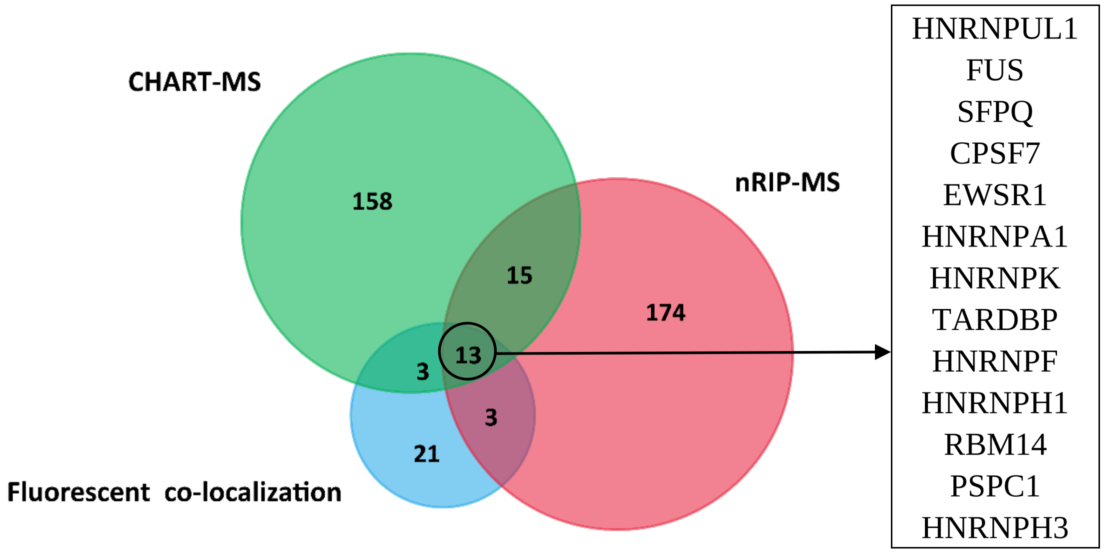

Dataset	Total number of gene symbols
nRIP-MS	205
Fluorescent co-localization (Naganuma et al., 2012)	189
CHRAT-MS (West et al., 2014)	40

Supplementary Figure S5. Comparison of NEAT1 nRIP-MS with previously published screening of paraspeckle proteins. Venn diagram comparing numbers of nRIP-MS identified paraspeckle proteins with previously published CHART-MS and Fluorescent co-localization screening methods. The overlapping proteins identified among all three methods were listed in the right inset.

>Universal KI template vector

ctaaattgtaagcgttaatattttgttaaaattcgcgttaaatttttgttaaatcagctcattttttaaccaataggccgaaatcggcaa
aatcccttataaatcaaaagaatagaccgagatagggttgagtgttgttccagtttggaacaagagtccactattaaagaacgtg
gactccaacgtcaaagggcgaaaaaccgtctatcagggcgatggcccactacgtgaaccatcaccctaatcaagttttttgggg
tcgaggtgccgtaaagcactaaatcggaaccctaaagggagcccccgatttagagcttgacggggaaagccggcgaacgtg
gcgagaaaggaagggaagaaagcgaaaggagcgggcgctagggcgctggcaagtgtagcggtcacgctgcgcgtaacc
accacacccgccgcgcttaatgcgccgctacagggcgcgtcccattcgccattcaggctgcgcaactgttgggaagggcgatc
ggtgcgggcctcttcgctattacgccagctggcgaaagggggatgtgctgcaaggcgattaagttgggtaacgccagggtttt
cccagtcacgacgttgtaaaacgacggccagtgagcgcgcgtaatacgactcactatagggcgaattgggtaccgggccccc
cctcgaggtcgacggtatcgataagcttagctgtaggacgtgcacccaggactcggctcacacatgcggatcctaaggtaccta
attgcctagaaaacatgaggatcacccatgtctgcaggtcgactctagaaaacatgaggatcacccatgtctgcagtattcccgg
gttcattagatcctaaggtacctaattgcctagaaaacatgaggatcacccatgtctgcaggtcgactctagaaaacatgaggat
cacccatgtctgcagtattcccgggttcattagatcctaaggtacctaattgcctagaaaacatgaggatcacccatgtctgcagg
tcgactccagaaaacatgaggatcacccatgtctgcagtattcccgggttcattagatcctaaggtacctaattgcctagaaaaca
tgaggatcacccatgtctgcaggtcgactctagaaaacatgaggatcacccatgtctgcagtattcccgggttcattagatcctaa
ggtacctaattgcctagaaaacatgaggatcacccatgtctgcaggtcgactctagaaaacatgaggatcacccatgtctgcagt
attcccgggttcattagatcctaaggtacctaattgcctagaaaacatgaggatcacccatgtctgcaggtcgactccagaaaac
atgaggatcacccatgtctgcagtattcccgggttcattagatcctaaggtacctaattgcctagaaaacatgaggatcacccatg
tctgcaggtcgactctagaaaacatgaggatcacccatgtctgcagtattcccgggttcattagatcctaaggtacctaattgccta
gaaaacatgaggatcacccatgtctgcaggtcgactctagaaaacatgaggatcacccatgtctgcagtattcccgggttcatta
gatcctaaggtacctaattgcctagaaaacatgaggatcacccatgtctgcaggtcgactccagaaaacatgaggatcacccat
gtctgcagtattcccgggttcattagatcctaaggtacctaattgcctagaaaacatgaggatcacccatgtctgcaggtcgactct
agaaaacatgaggatcacccatgtctgcagtattcccgggttcattagatcctaaggtacctaattgcctagaaaacatgaggat
cacccatgtctgcaggtcgactctagaaaacatgaggatcacccatgtctgcagtattcccgggttcattagatcctaaggtacct
aattgcctagaaaacatgaggatcacccatgtctgcaggtcgactccagaaaacatgaggatcacccatgtctgcagtattcccg
ggttcattagatctgcgcgcgatcgatagatcctaatcaacctctggattacaaaagcatcgtacgcgtacgtgtttggccccccc
ccctaacgttactggccgaagccgcttggaataaggccggtgtgcgtttgtctatatgttattttccaccatattgccgtcttttggc
aatgtgagggcccggaaacctggccctgtcttcttgacgagcattcctaggggtctttcccctctcgccaaaggaatgcaaggtc
tgttgaatgtcgtgaaggaagcagttcctctggaagcttcttgaagacaaacaacgtctgtagcgaccctttgcaggcagcgga
accccccacctggcgacaggtgcctctgcggccaaaagccacgtgtataagatacacctgcaaaggcggcacaaccccagtg
ccacgttgtgagttggatagttgtggaaagagtcaaatggctctcctcaagcgtattcaacaaggggctgaaggatgcccaga
aggtaccccattgtatgggatctgatctggggcctcggtgcacatgctttacatgtgtttagtcgaggttaaaaaaacgtctaggc
cccccgaaccacggggacgtggttttcctttgaaaaacacgatgataatatggccacaaccatggtgagcaagggcgaggag
ctgttcaccggggtggtgcccatcctggtcgagctggacggcgacgtaaacggccacaagttcagcgtgtccggcgagggc
gagggcgatgccacctacggcaagctgaccctgaagttcatctgcaccaccggcaagctgcccgtgccctggcccaccctcgt
gaccaccctgacctacggcgtgcagtgcttcagccgctaccccgaccacatgaagcagcacgacttcttcaagtccgccatgcc
cgaaggctacgtccaggagcgcaccatcttcttcaaggacgacggcaactacaagacccgcgccgaggtgaagttcgaggg
cgacaccctggtgaaccgcatcgagctgaagggcatcgacttcaaggaggacggcaacatcctggggcacaagctggagta
caactacaacagccacaacgtctatatcatggccgacaagcagaagaacggcatcaaggtgaacttcaagatccgccacaaca
tcgaggacggcagcgtgcagctcgccgaccactaccagcagaacacccccatcggcgacggccccgtgctgctgcccgaca
accactacctgagcacccagtccgccctgagcaaagaccccaacgagaagcgcgatcacatggtcctgctggagttcgtgacc
gccgccgggatcactctcggcatggacgagctgtacaagtaacccaaacctaccctctgcagcccgggggatccactagttcta
gagcggccgccaccgcggtggagctccagcttttgttccctttagtgagggttaattgcgcgcttggcgtaatcatggtcatagc
tgtttcctgtgtgaaattgttatccgctcacaattccacacaacatacgagccggaagcataaagtgtaaagcctggggtgcctaa

tgagtgagctaactcacattaattgcgttgcgctcactgcccgctttccagtcgggaaacctgtcgtgccagctgcattaatgaatc
ggccaacgcgcggggagaggcggtttgcgtattgggcgctcttccgcttcctcgctcactgactcgctgcgctcggtcgttcgg
ctgcggcgagcggtatcagctcactcaaaggcggtaatacggttatccacagaatcaggggataacgcaggaaagaacatgt
gagcaaaaggccagcaaaaggccaggaaccgtaaaaaggccgcgttgctggcgtttttccataggctccgcccccctgacga
gcatcacaaaaatcgacgctcaagtcagaggtggcgaaacccgacaggactataaagataccaggcgtttccccctggaagc
tccctcgtgcgctctcctgttccgaccctgccgcttaccggatacctgtccgcctttctcccttcgggaagcgtggcgctttctcata
gctcacgctgtaggtatctcagttcggtgtaggtcgttcgctccaagctgggctgtgtgcacgaaccccccgttcagcccgaccg
ctgcgccttatccggtaactatcgtcttgagtccaacccggtaagacacgacttatcgccactggcagcagccactggtaacagg
attagcagagcgaggtatgtaggcggtgctacagagttcttgaagtggtggcctaactacggctacactagaaggacagtattt
ggtatctgcgctctgctgaagccagttaccttcggaaaaagagttggtagctcttgatccggcaaacaaaccaccgctggtagc
ggtggtttttttgtttgcaagcagcagattacgcgcagaaaaaaaggatctcaagaagatcctttgatcttttctacggggtctgac
gctcagtggaacgaaaactcacgttaagggattttggtcatgagattatcaaaaaggatcttcacctagatccttttaaattaaaaa
tgaagttttaaatcaatctaaagtatatatgagtaaacttggtctgacagttaccaatgcttaatcagtgaggcacctatctcagcg
atctgtctatttcgttcatccatagttgcctgactccccgtcgtgtagataactacgatacgggagggcttaccatctggccccagt
gctgcaatgataccgcgagacccacgctcaccggctccagatttatcagcaataaaccagccagccggaagggccgagcgca
gaagtggtcctgcaactttatccgcctccatccagtctattaattgttgccgggaagctagagtaagtagttcgccagttaatagttt
gcgcaacgttgttgccattgctacaggcatcgtggtgtcacgctcgtcgtttggtatggcttcattcagctccggttcccaacgatc
aaggcgagttacatgatcccccatgttgtgcaaaaaagcggttagctccttcggtcctccgatcgttgtcagaagtaagttggcc
gcagtgttatcactcatggttatggcagcactgcataattctcttactgtcatgccatccgtaagatgcttttctgtgactggtgagta
ctcaaccaagtcattctgagaatagtgtatgcggcgaccgagttgctcttgcccggcgtcaatacgggataataccgcgccacat
agcagaactttaaaagtgctcatcattggaaaacgttcttcggggcgaaaactctcaaggatcttaccgctgttgagatccagttc
gatgtaacccactcgtgcacccaactgatcttcagcatcttttactttcaccagcgtttctgggtgagcaaaaacaggaaggcaaa
atgccgcaaaaaagggaataagggcgacacggaaatgttgaatactcatactcttcctttttcaatattattgaagcatttatcagg
gttattgtctcatgagcggatacatatttgaatgtatttagaaaaataaacaaataggggttccgcgcacatttccccgaaaagtg
ccac

Supplementary Figure 6. Full plasmid sequences of the Universal KI template vector. Purple, orange, blue, green and red letters indicate the sequences of 24×MS2, pre-gRNA, IRES, GFP and the CC-rich region, respectively.

>NEAT1-g1-KI

cacatctggatcagttcctccccattttgttggtcaaatccgatctgccatatcctgtgtaatgacaagtgagttgcattctcacc
gtcactcctggggtctctccgcttcccctgagctggctcagcagtctgctccatgtgttttgatgcagggtgacccattggtatt
cccgacactaacgcccccgtctgtggactgcttgctgcttgggcttcactgtgtctggtgttgacagtgcagacctaaaggtg
tgcacacatgtgcacacacactccgctgtcttcttgtttgcactggacttaaatatctatgagggttattttcaactgctgaatttg
gaatgatttttatatcttttctgctttctgcccatgtacatgtgtttattttacactgttgtgattggtagttactatgtggggacacaat
tacttgggctgaaataatccacctgttgtggttggggtcctctggggcattccagggtgagaggttgtcactgccacctgggc
catgtgggccggcaccagcattttgtggttacgaattctacagtcacaaatatctttgggcaaatccccttctatacctcaagg
cagcttttggtttgcaaccccactggccagagggaagggccagtcacttggctctctcactgccctgcgccccagatggttc
tagggctgctgttttcccttggccctgccaacaccactgtttttacttctgctcattggctgagtgcagtggttcctggaagcca
gtggcacgtttccccgcgtagctcgcttatcccacagcacacacccaagggttctgttgctaacacgctgaattaattctttgc
tcatcttacagagtgtgttttgactgcccccatttctgaggccttgtaaggccagagctttgttgcttcatcggcaggttgggac
ttagatggccgtgaatgtttcctctctgctgctgcagtaagtaagtgcccgcaccatagtgtgttagctgtaggacgtgcaccc
aggactcggctcacacatgcggatcctaaggtacctaattgcctagaaaacatgaggatcacccatgtctgcaggtcgact
ctagaaaacatgaggatcacccatgtctgcagtattcccgggttcattagatcctaaggtacctaattgcctagaaaacatga
ggatcacccatgtctgcaggtcgactctagaaaacatgaggatcacccatgtctgcagtattcccgggttcattagatcctaa
ggtacctaattgcctagaaaacatgaggatcacccatgtctgcaggtcgactccagaaaacatgaggatcacccatgtctgc
agtattcccgggttcattagatcctaaggtacctaattgcctagaaaacatgaggatcacccatgtctgcaggtcgactctag
aaaacatgaggatcacccatgtctgcagtattcccgggttcattagatcctaaggtacctaattgcctagaaaacatgaggat
cacccatgtctgcaggtcgactctagaaaacatgaggatcacccatgtctgcagtattcccgggttcattagatcctaaggta
cctaattgcctagaaaacatgaggatcacccatgtctgcaggtcgactccagaaaacatgaggatcacccatgtctgcagta
ttcccgggttcattagatcctaaggtacctaattgcctagaaaacatgaggatcacccatgtctgcaggtcgactctagaaaa
catgaggatcacccatgtctgcagtattcccgggttcattagatcctaaggtacctaattgcctagaaaacatgaggatcacc
catgtctgcaggtcgactctagaaaacatgaggatcacccatgtctgcagtattcccgggttcattagatcctaaggtaccta
attgcctagaaaacatgaggatcacccatgtctgcaggtcgactccagaaaacatgaggatcacccatgtctgcagtattcc
cgggttcattagatcctaaggtacctaattgcctagaaaacatgaggatcacccatgtctgcaggtcgactctagaaaacatg
aggatcacccatgtctgcagtattcccgggttcattagatcctaaggtacctaattgcctagaaaacatgaggatcacccatgt
ctgcaggtcgactctagaaaacatgaggatcacccatgtctgcagtattcccgggttcattagatcctaaggtacctaattgc
ctagaaaacatgaggatcacccatgtctgcaggtcgactccagaaaacatgaggatcacccatgtctgcagtattcccggg
ttcattagatctgcgcgcgatcgatagatcctaatcaacctctggattacaaaagcatcgtacgcgtacgtgtttggccccccc
ccctaacgttactggccgaagccgcttggaataaggccggtgtgcgtttgtctatatgttattttccaccatattgccgtcttttg
gcaatgtgagggcccggaaacctggccctgtcttcttgacgagcattcctaggggtctttcccctctcgccaaaggaatgca
aggtctgttgaatgtcgtgaaggaagcagttcctctggaagcttcttgaagacaaacaacgtctgtagcgaccctttgcagg
cagcggaaccccccacctggcgacaggtgcctctgcggccaaaagccacgtgtataagatacacctgcaaaggcggca
caaccccagtgccacgttgtgagttggatagttgtggaaagagtcaaatggctctcctcaagcgtattcaacaaggggctga
aggatgcccagaaggtaccccattgtatgggatctgatctggggcctcggtgcacatgctttacatgtgtttagtcgaggtta
aaaaaacgtctaggccccccgaaccacggggacgtggttttcctttgaaaaacacgatgataatatggccacaaccatggt
gagcaagggcgaggagctgttcaccggggtggtgcccatcctggtcgagctggacggcgacgtaaacggccacaagtt
cagcgtgtccggcgagggcgagggcgatgccacctacggcaagctgaccctgaagttcatctgcaccaccggcaagct
gcccgtgccctggcccaccctcgtgaccaccctgacctacggcgtgcagtgcttcagccgctaccccgaccacatgaagc
agcacgacttcttcaagtccgccatgcccgaaggctacgtccaggagcgcaccatcttcttcaaggacgacggcaactac
aagacccgcgccgaggtgaagttcgagggcgacaccctggtgaaccgcatcgagctgaagggcatcgacttcaaggag
gacggcaacatcctggggcacaagctggagtacaactacaacagccacaacgtctatatcatggccgacaagcagaaga
acggcatcaaggtgaacttcaagatccgccacaacatcgaggacggcagcgtgcagctcgccgaccactaccagcaga

acacccccatcggcgacggccccgtgctgctgcccgacaaccactacctgagcacccagtccgccctgagcaaagacc
ccaacgagaagcgcgatcacatggtcctgctggagttcgtgaccgccgccgggatcactctcggcatggacgagctgtac
aagtaacccaaacctacccttggaggctgaagttgaagcgaggctgtgaggggagatggacgtgtgaggagggatgatg
gggcttgagcaaagtgggggagggggcaaaggcagttggcccaacacattccccacccctttgagaggtctgaggcctg
cagacctggctcggagcccacctggtagtcctcagactgtgtgtgtgtgtgtgtgtgtgtgtgtgtgtgtgtgtgtgtgt
gtgtgtgtgtgtaaaagagagaagttgtggagaaatggggggctgattctgctcagattcatcaggatgagtagaaggcac
ccagctctcaccctggcctgacatgtgtgtccctgagcaggttacagtcctctctgagcctctgcttcccatctggaccctgct
gggcagggcttctgagctccttagcactagcaggaggggctccaggggccctccctccatggcagccaggacaggactc
tcaaatgaggacagcagagctcgtggggggctcccacggacccgccgtgggcccaggggaggcagagcctgagcca
acagcagtggtgctgtggaccgtggatcctgagggtggcctggggcaagtaccggctgagggtccaggtgggctttgtgt
acctttgggtcctggggccctggtgacttggactccaggttagagtcaagtgacaggagaaaggctggtggggccctgtg
cttccgacttcatttcgagtgatggcagttcccaggaaggaatccacagctgacggtggctgacagatcagagaatggaag
gcgaggcaggcgggcgtctgcgtgacctcaggtgcttggggcccagcagacccagagaaccatttccactaggccagg
gtgccggaagtgtccacaggtcttagattccctgttcagatgaaaagatttgtgcctttaatgataaaagtgatctgcatagagt
caaaaattcaagccatgggtataaaatgcaagtaaaatccctgccctcacctatcccaccctactacacagagatgtcctctc
gagtttcctagactcactctggaaatttctgtatacacacagaagcttgtgcctctgctcgtgaaggcagagggagggagag
ctgaagggccagcaccttctcacctgtgggccccctcagtgctcggtcccagagcatgcaggactgtgcctcgtgttcagtt
tgctggtctgacttcatgctccttgggcaggatatgcatgtgccatgctaggagacatgtggatgtgaagctgggggacaat
gtcccctggctatgcctttacagggaagtaaggaaggtaggaggtgagcctgggagggagggagggaggcgcggag
ccgccgcaggtgtttcttttactgagtgcagcccatggccgcactcaggttttgcttttcaccttcccatctgtgaaagagtgag
caggaaaaagcaaaa

Supplementary Figure 7. Genomic sequence of the NEAT1 locus after KI by gRNA-1. Black, purple, orange, blue, green and red letters indicate the sequences of NEAT1 (partial), 24×MS2, pre-gRNA, IRES, GFP and the CC-rich region, respectively. The yellow highlights indicate the MHAs of the MMEJ-repair. The underline shows the newly fusion gRNA (black) and the corresponding PAM motif (red).

>NEAT1-g2-KI

ggggagatggacgtgtgaggagggatgatggggcttgagcaaagtgggggagggggcaaaggcagttggcccaaca
cattccccaccccctttgagaggtctgaggcctgcagacctggctcggagcccacctggtagtcctcagactgtgtgtgtgtg
tgtgtgtgtgtgtgtgtgtgtgtgtgtgtgtgtgtgtgtgtgtgtaaaagagagaagttgtggagaaatggggggctgattct
gctcagattcatcaggatgagtagaaggcacccagctctcaccctggcctgacatgtgtgtccctgagcaggttacagtcct
ctctgagcctctgcttcccatctggaccctgctgggcagggcttctgagctccttagcactagcaggaggggctccagggg
ccctccctccatggcagccaggacaggactctcaaatgaggacagcagagctcgtggggggctcccacggacccgccg
tgggcccaggggaggcagagcctgagccaacagcagtggtgctgtggaccgtggatcctgagggtggcctggggcaa
gtaccggctgagggtccaggtgggctttgtgtacctttgggtcctggggccctggtgacttggactccaggttagagtcaag
tgacaggagaaaggctggtggggccctgtgcttccgacttcatttcgagtgatggcagttcccaggaaggaatccacagct
gacggtggctgacagatcagagaatggaaggcgaggcaggcgggcgtctgcgtgacctcaggtgcttggggcccagc
agacccagagaaccatttccactaggccagggtgccggaagtgtccacaggtcttagattccctgttcagatgaaaagattt
gtgcctttaatgataaaagtgatctgcatagagtcaaaaattcaagccatgggtataaaatgcaagtaaaatccctgccctca
cctatcccaccctactacacagagatgtcctctcagctgtaggacgtgcacccaggactcggctcacacatgcggatccta
aggtacctaattgcctagaaaacatgaggatcacccatgtctgcaggtcgactctagaaaacatgaggatcacccatgtctg
cagtattcccgggttcattagatcctaaggtacctaattgcctagaaaacatgaggatcacccatgtctgcaggtcgactcta
gaaaacatgaggatcacccatgtctgcagtattcccgggttcattagatcctaaggtacctaattgcctagaaaacatgagga
tcacccatgtctgcaggtcgactccagaaaacatgaggatcacccatgtctgcagtattcccgggttcattagatcctaaggt
acctaattgcctagaaaacatgaggatcacccatgtctgcaggtcgactctagaaaacatgaggatcacccatgtctgcagt
attcccgggttcattagatcctaaggtacctaattgcctagaaaacatgaggatcacccatgtctgcaggtcgactctagaaa
acatgaggatcacccatgtctgcagtattcccgggttcattagatcctaaggtacctaattgcctagaaaacatgaggatcac
ccatgtctgcaggtcgactccagaaaacatgaggatcacccatgtctgcagtattcccgggttcattagatcctaaggtacct
aattgcctagaaaacatgaggatcacccatgtctgcaggtcgactctagaaaacatgaggatcacccatgtctgcagtattc
ccgggttcattagatcctaaggtacctaattgcctagaaaacatgaggatcacccatgtctgcaggtcgactctagaaaacat
gaggatcacccatgtctgcagtattcccgggttcattagatcctaaggtacctaattgcctagaaaacatgaggatcacccat
gtctgcaggtcgactccagaaaacatgaggatcacccatgtctgcagtattcccgggttcattagatcctaaggtacctaatt
gcctagaaaacatgaggatcacccatgtctgcaggtcgactctagaaaacatgaggatcacccatgtctgcagtattcccg
ggttcattagatcctaaggtacctaattgcctagaaaacatgaggatcacccatgtctgcaggtcgactctagaaaacatgag
gatcacccatgtctgcagtattcccgggttcattagatcctaaggtacctaattgcctagaaaacatgaggatcacccatgtct
gcaggtcgactccagaaaacatgaggatcacccatgtctgcagtattcccgggttcattagatctgcgcgcgatcgatagat
cctaatcaacctctggattacaaaagcatcgtacgcgtacgtgtttggcccccccccctaacgttactggccgaagccgctt
ggaataaggccggtgtgcgtttgtctatatgttattttccaccatattgccgtcttttggcaatgtgagggcccggaaacctggc
cctgtcttcttgacgagcattcctaggggtctttcccctctcgccaaaggaatgcaaggtctgttgaatgtcgtgaaggaagc
agttcctctggaagcttcttgaagacaaacaacgtctgtagcgaccctttgcaggcagcggaaccccccacctggcgacag
gtgcctctgcggccaaaagccacgtgtataagatacacctgcaaaggcggcacaaccccagtgccacgttgtgagttgga
tagttgtggaaagagtcaaatggctctcctcaagcgtattcaacaaggggctgaaggatgcccagaaggtaccccattgtat
gggatctgatctggggcctcggtgcacatgctttacatgtgtttagtcgaggttaaaaaaacgtctaggccccccgaaccac
ggggacgtggttttcctttgaaaaacacgatgataatatggccacaaccatggtgagcaagggcgaggagctgttcaccgg
ggtggtgcccatcctggtcgagctggacggcgacgtaaacggccacaagttcagcgtgtccggcgagggcgagggcga
tgccacctacggcaagctgaccctgaagttcatctgcaccaccggcaagctgcccgtgccctggcccaccctcgtgacca
ccctgacctacggcgtgcagtgcttcagccgctaccccgaccacatgaagcagcacgacttcttcaagtccgccatgccc
gaaggctacgtccaggagcgcaccatcttcttcaaggacgacggcaactacaagacccgcgccgaggtgaagttcgagg
gcgacaccctggtgaaccgcatcgagctgaagggcatcgacttcaaggaggacggcaacatcctggggcacaagctgg
agtacaactacaacagccacaacgtctatatcatggccgacaagcagaagaacggcatcaaggtgaacttcaagatccgc

cacaacatcgaggacggcagcgtgcagctcgccgaccactaccagcagaacacccccatcggcgacggccccgtgct
gctgcccgacaaccactacctgagcacccagtccgccctgagcaaagaccccaacgagaagcgcgatcacatggtcctg
ctggagttcgtgaccgccgccgggatcactctcggcatggacgagctgtacaagtaacccaaacctaccctgagtttccta
gactcactctggaaatttctgtatacacacagaagcttgtgcctctgctcgtgaaggcagagggagggagagctgaagggc
cagcaccttctcacctgtgggccccctcagtgctcggtcccagagcatgcaggactgtgcctcgtgttcagtttgctggtctg
acttcatgctccttgggcaggatatgcatgtgccatgctaggagacatgtggatgtgaagctgggggacaatgtcccctggc
tatgcctttacaagggaagtaaggaaggtaggaggtgagcctgggagggagggagggaggcgcggagccgccgcag
gtgtttcttttactgagtgcagcccatggccgcactcaggttttgcttttcaccttcccatctgtgaaagagtgagcaggaaaaa
gcaaaa

Supplementary Figure 8. Genomic sequence of the NEAT1 locus after KI by gRNA-2. Black, purple, orange, blue, green and red letters indicate the sequences of NEAT1 (partial), 24×MS2, pre-gRNA, IRES, GFP and the CC-rich region, respectively. The yellow highlights indicate the MHAs of the MMEJ-repair. The underline shows the newly fusion gRNA (black) and the corresponding PAM motif (red).
