# Supplementary material for: Live cell imaging and proteomic profiling of endogenous NEAT1 lncRNA by CRISPR/Cas9-mediated knock-in
Source: Protein Cell. 2020 May 26;11(9):641–60. doi: 10.1007/s13238-020-00706-w (PMC7452982; doi:10.1007/s13238-020-00706-w)
Supplement: Supplementary file 7 — Supplementary material 7 (DOCX 1998 kb) [file 13238_2020_706_MOESM7_ESM.docx]

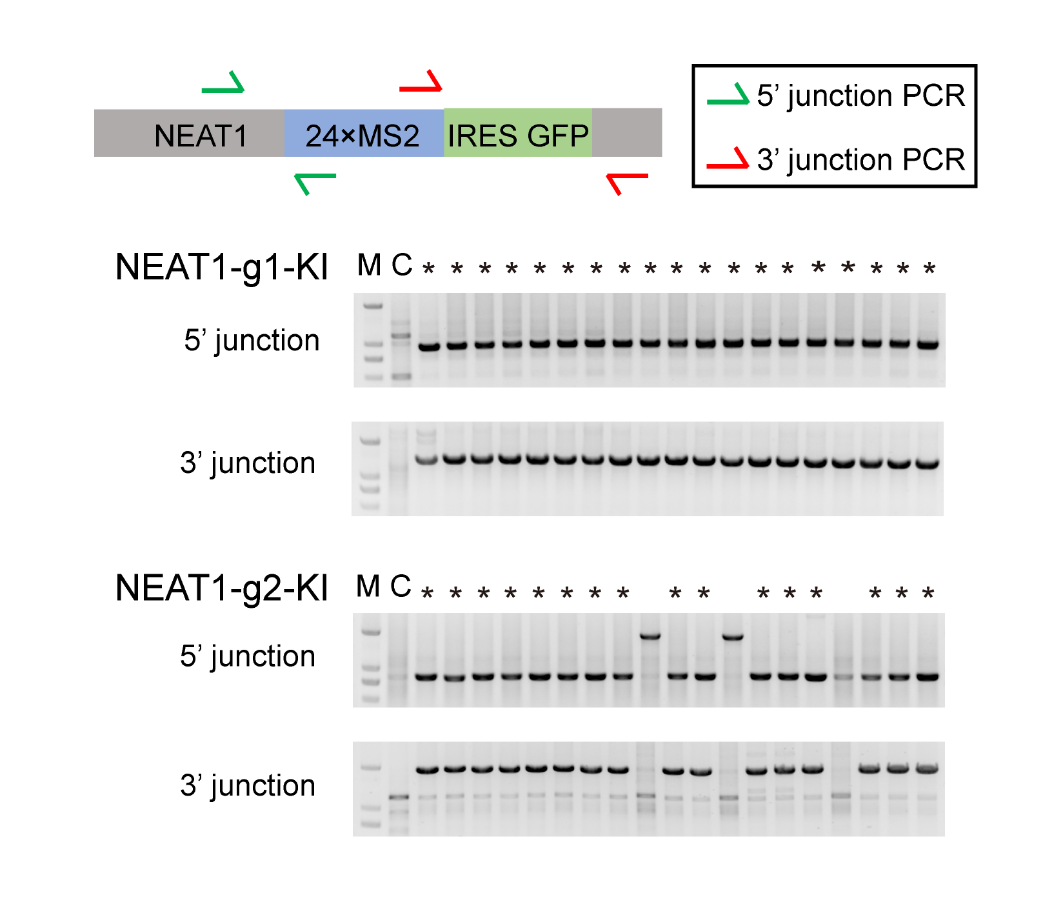


**Supplementary Figure S1. Junction PCR of the single clones from two independent KI cell lines.** Upper panel shows the schematic of the 5’ and 3’ junction PCR primers used for genotyping. Lower panel shows the junction PCR of the single clones of two KI cell lines. Expected band sizes from the knock-in alleles of line1 are around 0.9 KB for 5’ junction PCR and 1.4 KB for 3’ junction PCR, while line2 are around 0.6 KB for 5’ junction PCR and 1.9 KB for 3’ junction PCR. WT HEK293T gDNA was used as a negative control. C indicates WT control, * indicate single clones with expected band size.

**
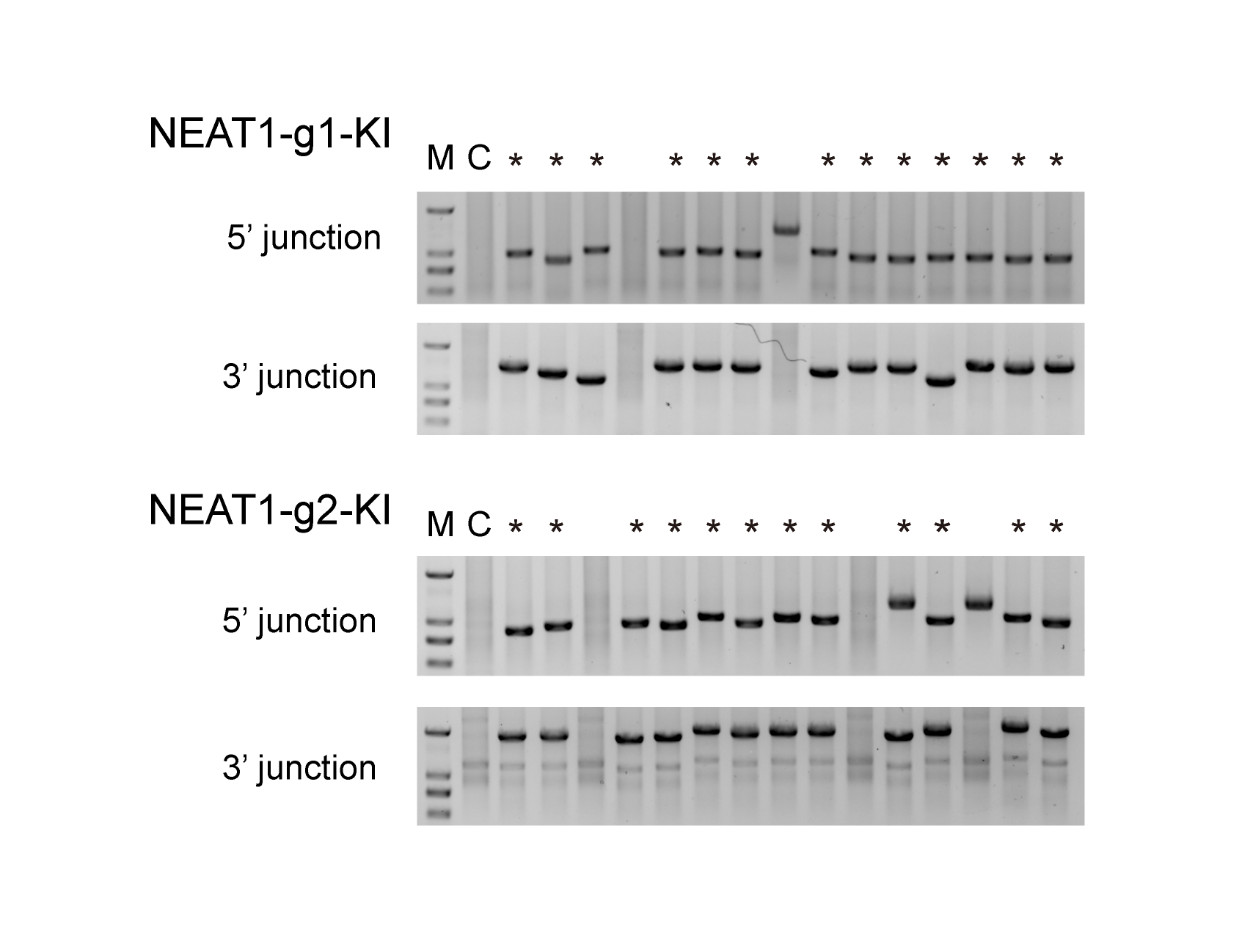
**

**Supplementary Figure S2. Genotyping PCR of the GFP positive subclones from two NEAT1 KI cell lines after paired gRNAs KO.** 5’ and 3’ junction PCR were carried out for the GFP positive subclones of two NEAT1 KI cell lines after double KO of IRES-GFP fragment. On-target KI cells without simultaneous cutting of paired gRNAs would still retain the expected band sizes of PCR products and GFP expression, while off-target insertions could not generate the expected junction PCR products. WT HEK293T gDNA was used as a negative control. C indicates WT control, * indicate single clones with expected band size.


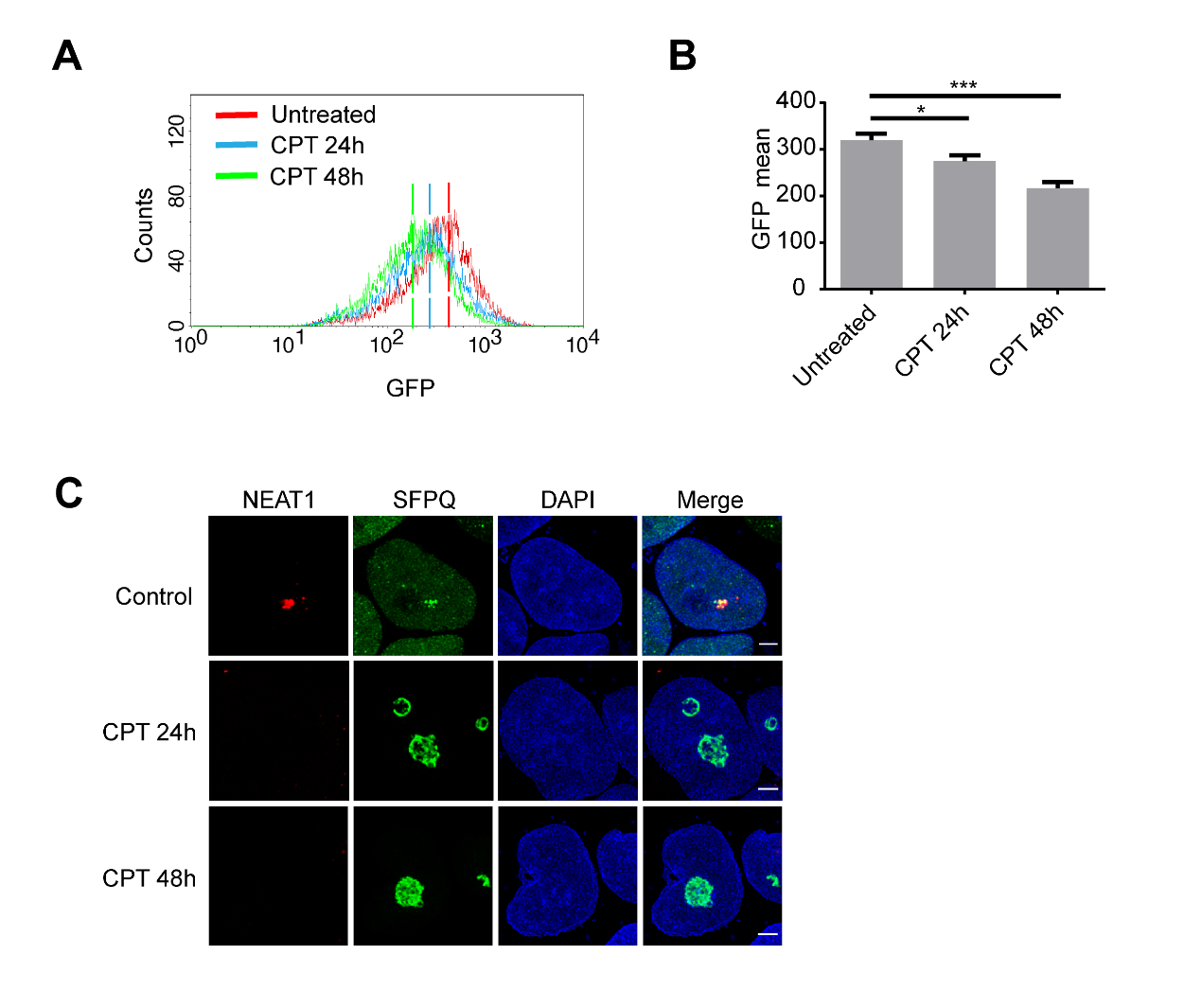


**Supplementary Figure S3. GFP intensity of CERTIS labeled NEAT1 correctly mirror the regulatory relationship by CPT in live cells**

1. GFP peak shifted to the left gradually after 5 μM CPT treatment for 24 and 48 h, respectively. Untreated cells were used as control. GFP fluorescence intensity was detected by flow cytometer and the dotted lines indicated the mean of GFP fluorescence intensity.
2. Mean fluorescence intensity (from A) showed time-dependent decrease of NEAT1 level after CPT treatment for 24 and 48 h.
3. NEAT1 (red) disaggregated and SFPQ relocated to the nucleolus after CPT treatment. Scale bar: 2 μm.


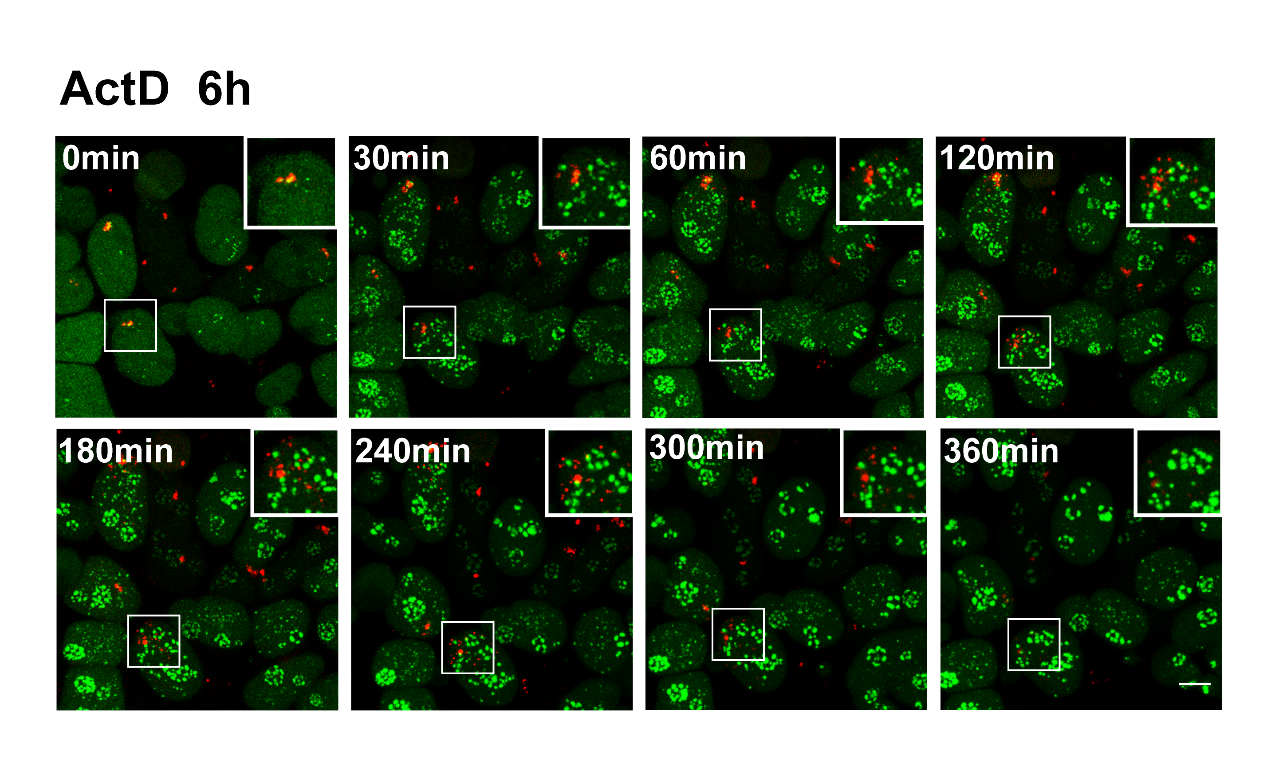


**Supplementary Figure S4. CERTIS enables long-term time-lapse imaging of endogenous NEAT1 in living cells.** Snapshots displayed the dynamic of NEAT1 and SFPQ after 250 ng/ml ActD treatment for 6 h, with a 15 min time-lapse. Insets showed the magnified boxed region of the merged channel of NEAT1 and SFPQ. Scale bar: 2 μm.

| HNRNPUL1  FUS  SFPQ  CPSF7  EWSR1  HNRNPA1  HNRNPK TARDBP  HNRNPF  HNRNPH1  RBM14  PSPC1  HNRNPH3 |
| --- |


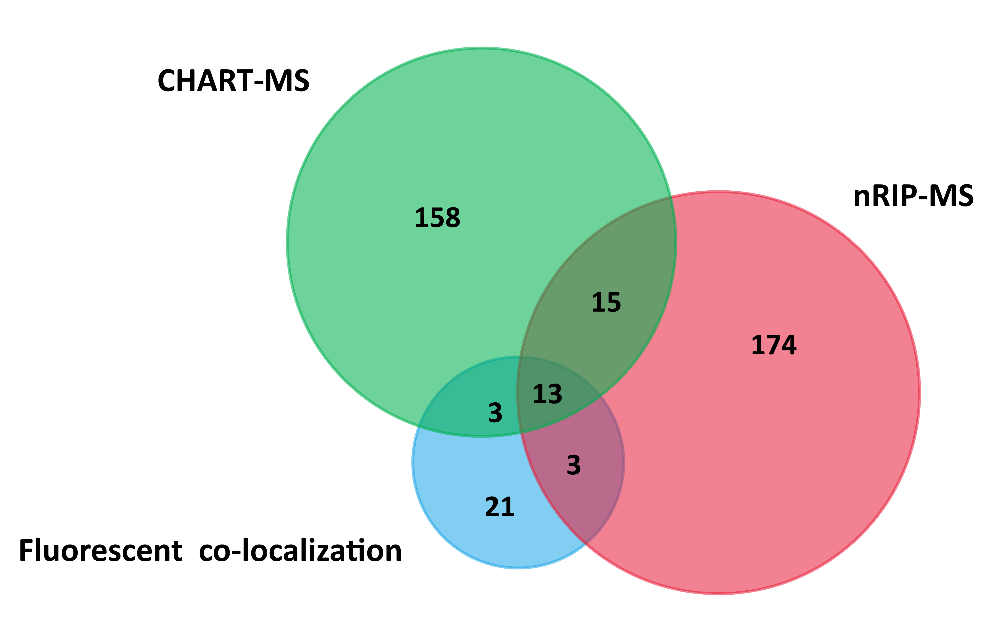


| Dataset | Total number of gene symbols |
| --- | --- |
| nRIP-MS | 205 |
| Fluorescent co-localization (Naganuma et al., 2012) | 189 |
| CHRAT-MS (West et al., 2014) | 40 |

**Supplementary Figure S5. Comparison of NEAT1** **nRIP-MS with** **previously published screening of paraspeckle proteins.** Venn diagram comparing numbers of nRIP-MS identified paraspeckle proteins with previously published CHART-MS and Fluorescent co-localization screening methods. The overlapping proteins identified among all three methods were listed in the right inset.

**>****Universal KI template vector**

ctaaattgtaagcgttaatattttgttaaaattcgcgttaaatttttgttaaatcagctcattttttaaccaataggccgaaatcggcaaaatcccttataaatcaaaagaatagaccgagatagggttgagtgttgttccagtttggaacaagagtccactattaaagaacgtggactccaacgtcaaagggcgaaaaaccgtctatcagggcgatggcccactacgtgaaccatcaccctaatcaagttttttggggtcgaggtgccgtaaagcactaaatcggaaccctaaagggagcccccgatttagagcttgacggggaaagccggcgaacgtggcgagaaaggaagggaagaaagcgaaaggagcgggcgctagggcgctggcaagtgtagcggtcacgctgcgcgtaaccaccacacccgccgcgcttaatgcgccgctacagggcgcgtcccattcgccattcaggctgcgcaactgttgggaagggcgatcggtgcgggcctcttcgctattacgccagctggcgaaagggggatgtgctgcaaggcgattaagttgggtaacgccagggttttcccagtcacgacgttgtaaaacgacggccagtgagcgcgcgtaatacgactcactatagggcgaattgggtaccgggccccccctcgaggtcgacggtatcgataagcttagctgtaggacgtgcacccaggactcggctcacacatgcggatcctaaggtacctaattgcctagaaaacatgaggatcacccatgtctgcaggtcgactctagaaaacatgaggatcacccatgtctgcagtattcccgggttcattagatcctaaggtacctaattgcctagaaaacatgaggatcacccatgtctgcaggtcgactctagaaaacatgaggatcacccatgtctgcagtattcccgggttcattagatcctaaggtacctaattgcctagaaaacatgaggatcacccatgtctgcaggtcgactccagaaaacatgaggatcacccatgtctgcagtattcccgggttcattagatcctaaggtacctaattgcctagaaaacatgaggatcacccatgtctgcaggtcgactctagaaaacatgaggatcacccatgtctgcagtattcccgggttcattagatcctaaggtacctaattgcctagaaaacatgaggatcacccatgtctgcaggtcgactctagaaaacatgaggatcacccatgtctgcagtattcccgggttcattagatcctaaggtacctaattgcctagaaaacatgaggatcacccatgtctgcaggtcgactccagaaaacatgaggatcacccatgtctgcagtattcccgggttcattagatcctaaggtacctaattgcctagaaaacatgaggatcacccatgtctgcaggtcgactctagaaaacatgaggatcacccatgtctgcagtattcccgggttcattagatcctaaggtacctaattgcctagaaaacatgaggatcacccatgtctgcaggtcgactctagaaaacatgaggatcacccatgtctgcagtattcccgggttcattagatcctaaggtacctaattgcctagaaaacatgaggatcacccatgtctgcaggtcgactccagaaaacatgaggatcacccatgtctgcagtattcccgggttcattagatcctaaggtacctaattgcctagaaaacatgaggatcacccatgtctgcaggtcgactctagaaaacatgaggatcacccatgtctgcagtattcccgggttcattagatcctaaggtacctaattgcctagaaaacatgaggatcacccatgtctgcaggtcgactctagaaaacatgaggatcacccatgtctgcagtattcccgggttcattagatcctaaggtacctaattgcctagaaaacatgaggatcacccatgtctgcaggtcgactccagaaaacatgaggatcacccatgtctgcagtattcccgggttcattagatctgcgcgcgatcgatagatcctaatcaacctctggattacaaaagcatcgtacgcgtacgtgtttggcccccccccctaacgttactggccgaagccgcttggaataaggccggtgtgcgtttgtctatatgttattttccaccatattgccgtcttttggcaatgtgagggcccggaaacctggccctgtcttcttgacgagcattcctaggggtctttcccctctcgccaaaggaatgcaaggtctgttgaatgtcgtgaaggaagcagttcctctggaagcttcttgaagacaaacaacgtctgtagcgaccctttgcaggcagcggaaccccccacctggcgacaggtgcctctgcggccaaaagccacgtgtataagatacacctgcaaaggcggcacaaccccagtgccacgttgtgagttggatagttgtggaaagagtcaaatggctctcctcaagcgtattcaacaaggggctgaaggatgcccagaaggtaccccattgtatgggatctgatctggggcctcggtgcacatgctttacatgtgtttagtcgaggttaaaaaaacgtctaggccccccgaaccacggggacgtggttttcctttgaaaaacacgatgataatatggccacaaccatggtgagcaagggcgaggagctgttcaccggggtggtgcccatcctggtcgagctggacggcgacgtaaacggccacaagttcagcgtgtccggcgagggcgagggcgatgccacctacggcaagctgaccctgaagttcatctgcaccaccggcaagctgcccgtgccctggcccaccctcgtgaccaccctgacctacggcgtgcagtgcttcagccgctaccccgaccacatgaagcagcacgacttcttcaagtccgccatgcccgaaggctacgtccaggagcgcaccatcttcttcaaggacgacggcaactacaagacccgcgccgaggtgaagttcgagggcgacaccctggtgaaccgcatcgagctgaagggcatcgacttcaaggaggacggcaacatcctggggcacaagctggagtacaactacaacagccacaacgtctatatcatggccgacaagcagaagaacggcatcaaggtgaacttcaagatccgccacaacatcgaggacggcagcgtgcagctcgccgaccactaccagcagaacacccccatcggcgacggccccgtgctgctgcccgacaaccactacctgagcacccagtccgccctgagcaaagaccccaacgagaagcgcgatcacatggtcctgctggagttcgtgaccgccgccgggatcactctcggcatggacgagctgtacaagtaacccaaacctaccctctgcagcccgggggatccactagttctagagcggccgccaccgcggtggagctccagcttttgttccctttagtgagggttaattgcgcgcttggcgtaatcatggtcatagctgtttcctgtgtgaaattgttatccgctcacaattccacacaacatacgagccggaagcataaagtgtaaagcctggggtgcctaatgagtgagctaactcacattaattgcgttgcgctcactgcccgctttccagtcgggaaacctgtcgtgccagctgcattaatgaatcggccaacgcgcggggagaggcggtttgcgtattgggcgctcttccgcttcctcgctcactgactcgctgcgctcggtcgttcggctgcggcgagcggtatcagctcactcaaaggcggtaatacggttatccacagaatcaggggataacgcaggaaagaacatgtgagcaaaaggccagcaaaaggccaggaaccgtaaaaaggccgcgttgctggcgtttttccataggctccgcccccctgacgagcatcacaaaaatcgacgctcaagtcagaggtggcgaaacccgacaggactataaagataccaggcgtttccccctggaagctccctcgtgcgctctcctgttccgaccctgccgcttaccggatacctgtccgcctttctcccttcgggaagcgtggcgctttctcatagctcacgctgtaggtatctcagttcggtgtaggtcgttcgctccaagctgggctgtgtgcacgaaccccccgttcagcccgaccgctgcgccttatccggtaactatcgtcttgagtccaacccggtaagacacgacttatcgccactggcagcagccactggtaacaggattagcagagcgaggtatgtaggcggtgctacagagttcttgaagtggtggcctaactacggctacactagaaggacagtatttggtatctgcgctctgctgaagccagttaccttcggaaaaagagttggtagctcttgatccggcaaacaaaccaccgctggtagcggtggtttttttgtttgcaagcagcagattacgcgcagaaaaaaaggatctcaagaagatcctttgatcttttctacggggtctgacgctcagtggaacgaaaactcacgttaagggattttggtcatgagattatcaaaaaggatcttcacctagatccttttaaattaaaaatgaagttttaaatcaatctaaagtatatatgagtaaacttggtctgacagttaccaatgcttaatcagtgaggcacctatctcagcgatctgtctatttcgttcatccatagttgcctgactccccgtcgtgtagataactacgatacgggagggcttaccatctggccccagtgctgcaatgataccgcgagacccacgctcaccggctccagatttatcagcaataaaccagccagccggaagggccgagcgcagaagtggtcctgcaactttatccgcctccatccagtctattaattgttgccgggaagctagagtaagtagttcgccagttaatagtttgcgcaacgttgttgccattgctacaggcatcgtggtgtcacgctcgtcgtttggtatggcttcattcagctccggttcccaacgatcaaggcgagttacatgatcccccatgttgtgcaaaaaagcggttagctccttcggtcctccgatcgttgtcagaagtaagttggccgcagtgttatcactcatggttatggcagcactgcataattctcttactgtcatgccatccgtaagatgcttttctgtgactggtgagtactcaaccaagtcattctgagaatagtgtatgcggcgaccgagttgctcttgcccggcgtcaatacgggataataccgcgccacatagcagaactttaaaagtgctcatcattggaaaacgttcttcggggcgaaaactctcaaggatcttaccgctgttgagatccagttcgatgtaacccactcgtgcacccaactgatcttcagcatcttttactttcaccagcgtttctgggtgagcaaaaacaggaaggcaaaatgccgcaaaaaagggaataagggcgacacggaaatgttgaatactcatactcttcctttttcaatattattgaagcatttatcagggttattgtctcatgagcggatacatatttgaatgtatttagaaaaataaacaaataggggttccgcgcacatttccccgaaaagtgccac

**Supplementary Figure 6. Full plasmid sequences of the Universal KI template vector.** Purple, orange, blue, green and red letters indicate the sequences of 24×MS2, pre-gRNA, IRES, GFP and the CC-rich region, respectively.

**>NEAT1-g1-KI**

cacatctggatcagttcctccccattttgttggtcaaatccgatctgccatatcctgtgtaatgacaagtgagttgcattctcaccgtcactcctggggtctctccgcttcccctgagctggctcagcagtctgctccatgtgttttgatgcagggtgacccattggtattcccgacactaacgcccccgtctgtggactgcttgctgcttgggcttcactgtgtctggtgttgacagtgcagacctaaaggtgtgcacacatgtgcacacacactccgctgtcttcttgtttgcactggacttaaatatctatgagggttattttcaactgctgaatttggaatgatttttatatcttttctgctttctgcccatgtacatgtgtttattttacactgttgtgattggtagttactatgtggggacacaattacttgggctgaaataatccacctgttgtggttggggtcctctggggcattccagggtgagaggttgtcactgccacctgggccatgtgggccggcaccagcattttgtggttacgaattctacagtcacaaatatctttgggcaaatccccttctatacctcaaggcagcttttggtttgcaaccccactggccagagggaagggccagtcacttggctctctcactgccctgcgccccagatggttctagggctgctgttttcccttggccctgccaacaccactgtttttacttctgctcattggctgagtgcagtggttcctggaagccagtggcacgtttccccgcgtagctcgcttatcccacagcacacacccaagggttctgttgctaacacgctgaattaattctttgctcatcttacagagtgtgttttgactgcccccatttctgaggccttgtaaggccagagctttgttgcttcatcggcaggttgggacttagatggccgtgaatgtttcctctctgctgctgcagtaagtaagtgcccgcaccatagtgtgttagctgtaggacgtgcacccaggactcggctcacacatgcggatcctaaggtacctaattgcctagaaaacatgaggatcacccatgtctgcaggtcgactctagaaaacatgaggatcacccatgtctgcagtattcccgggttcattagatcctaaggtacctaattgcctagaaaacatgaggatcacccatgtctgcaggtcgactctagaaaacatgaggatcacccatgtctgcagtattcccgggttcattagatcctaaggtacctaattgcctagaaaacatgaggatcacccatgtctgcaggtcgactccagaaaacatgaggatcacccatgtctgcagtattcccgggttcattagatcctaaggtacctaattgcctagaaaacatgaggatcacccatgtctgcaggtcgactctagaaaacatgaggatcacccatgtctgcagtattcccgggttcattagatcctaaggtacctaattgcctagaaaacatgaggatcacccatgtctgcaggtcgactctagaaaacatgaggatcacccatgtctgcagtattcccgggttcattagatcctaaggtacctaattgcctagaaaacatgaggatcacccatgtctgcaggtcgactccagaaaacatgaggatcacccatgtctgcagtattcccgggttcattagatcctaaggtacctaattgcctagaaaacatgaggatcacccatgtctgcaggtcgactctagaaaacatgaggatcacccatgtctgcagtattcccgggttcattagatcctaaggtacctaattgcctagaaaacatgaggatcacccatgtctgcaggtcgactctagaaaacatgaggatcacccatgtctgcagtattcccgggttcattagatcctaaggtacctaattgcctagaaaacatgaggatcacccatgtctgcaggtcgactccagaaaacatgaggatcacccatgtctgcagtattcccgggttcattagatcctaaggtacctaattgcctagaaaacatgaggatcacccatgtctgcaggtcgactctagaaaacatgaggatcacccatgtctgcagtattcccgggttcattagatcctaaggtacctaattgcctagaaaacatgaggatcacccatgtctgcaggtcgactctagaaaacatgaggatcacccatgtctgcagtattcccgggttcattagatcctaaggtacctaattgcctagaaaacatgaggatcacccatgtctgcaggtcgactccagaaaacatgaggatcacccatgtctgcagtattcccgggttcattagatctgcgcgcgatcgatagatcctaatcaacctctggattacaaaagcatcgtacgcgtacgtgtttggcccccccccctaacgttactggccgaagccgcttggaataaggccggtgtgcgtttgtctatatgttattttccaccatattgccgtcttttggcaatgtgagggcccggaaacctggccctgtcttcttgacgagcattcctaggggtctttcccctctcgccaaaggaatgcaaggtctgttgaatgtcgtgaaggaagcagttcctctggaagcttcttgaagacaaacaacgtctgtagcgaccctttgcaggcagcggaaccccccacctggcgacaggtgcctctgcggccaaaagccacgtgtataagatacacctgcaaaggcggcacaaccccagtgccacgttgtgagttggatagttgtggaaagagtcaaatggctctcctcaagcgtattcaacaaggggctgaaggatgcccagaaggtaccccattgtatgggatctgatctggggcctcggtgcacatgctttacatgtgtttagtcgaggttaaaaaaacgtctaggccccccgaaccacggggacgtggttttcctttgaaaaacacgatgataatatggccacaaccatggtgagcaagggcgaggagctgttcaccggggtggtgcccatcctggtcgagctggacggcgacgtaaacggccacaagttcagcgtgtccggcgagggcgagggcgatgccacctacggcaagctgaccctgaagttcatctgcaccaccggcaagctgcccgtgccctggcccaccctcgtgaccaccctgacctacggcgtgcagtgcttcagccgctaccccgaccacatgaagcagcacgacttcttcaagtccgccatgcccgaaggctacgtccaggagcgcaccatcttcttcaaggacgacggcaactacaagacccgcgccgaggtgaagttcgagggcgacaccctggtgaaccgcatcgagctgaagggcatcgacttcaaggaggacggcaacatcctggggcacaagctggagtacaactacaacagccacaacgtctatatcatggccgacaagcagaagaacggcatcaaggtgaacttcaagatccgccacaacatcgaggacggcagcgtgcagctcgccgaccactaccagcagaacacccccatcggcgacggccccgtgctgctgcccgacaaccactacctgagcacccagtccgccctgagcaaagaccccaacgagaagcgcgatcacatggtcctgctggagttcgtgaccgccgccgggatcactctcggcatggacgagctgtacaagtaacccaaacctacccttggaggctgaagttgaagcgaggctgtgaggggagatggacgtgtgaggagggatgatggggcttgagcaaagtgggggagggggcaaaggcagttggcccaacacattccccacccctttgagaggtctgaggcctgcagacctggctcggagcccacctggtagtcctcagactgtgtgtgtgtgtgtgtgtgtgtgtgtgtgtgtgtgtgtgtgtgtgtgtgtgtgtgtgtaaaagagagaagttgtggagaaatggggggctgattctgctcagattcatcaggatgagtagaaggcacccagctctcaccctggcctgacatgtgtgtccctgagcaggttacagtcctctctgagcctctgcttcccatctggaccctgctgggcagggcttctgagctccttagcactagcaggaggggctccaggggccctccctccatggcagccaggacaggactctcaaatgaggacagcagagctcgtggggggctcccacggacccgccgtgggcccaggggaggcagagcctgagccaacagcagtggtgctgtggaccgtggatcctgagggtggcctggggcaagtaccggctgagggtccaggtgggctttgtgtacctttgggtcctggggccctggtgacttggactccaggttagagtcaagtgacaggagaaaggctggtggggccctgtgcttccgacttcatttcgagtgatggcagttcccaggaaggaatccacagctgacggtggctgacagatcagagaatggaaggcgaggcaggcgggcgtctgcgtgacctcaggtgcttggggcccagcagacccagagaaccatttccactaggccagggtgccggaagtgtccacaggtcttagattccctgttcagatgaaaagatttgtgcctttaatgataaaagtgatctgcatagagtcaaaaattcaagccatgggtataaaatgcaagtaaaatccctgccctcacctatcccaccctactacacagagatgtcctctcgagtttcctagactcactctggaaatttctgtatacacacagaagcttgtgcctctgctcgtgaaggcagagggagggagagctgaagggccagcaccttctcacctgtgggccccctcagtgctcggtcccagagcatgcaggactgtgcctcgtgttcagtttgctggtctgacttcatgctccttgggcaggatatgcatgtgccatgctaggagacatgtggatgtgaagctgggggacaatgtcccctggctatgcctttacaagggaagtaaggaaggtaggaggtgagcctgggagggagggagggaggcgcggagccgccgcaggtgtttcttttactgagtgcagcccatggccgcactcaggttttgcttttcaccttcccatctgtgaaagagtgagcaggaaaaagcaaaa

**Supplementary Figure 7. Genomic sequence of the NEAT1 locus after KI by gRNA-1.** Black, purple, orange, blue, green and red letters indicate the sequences of NEAT1 (partial), 24×MS2, pre-gRNA, IRES, GFP and the CC-rich region, respectively. The yellow highlights indicate the MHAs of the MMEJ-repair. The underline shows the newly fusion gRNA (black) and the corresponding PAM motif (red).

**>NEAT1-g2-KI**

ggggagatggacgtgtgaggagggatgatggggcttgagcaaagtgggggagggggcaaaggcagttggcccaacacattccccacccctttgagaggtctgaggcctgcagacctggctcggagcccacctggtagtcctcagactgtgtgtgtgtgtgtgtgtgtgtgtgtgtgtgtgtgtgtgtgtgtgtgtgtgtgtgtgtaaaagagagaagttgtggagaaatggggggctgattctgctcagattcatcaggatgagtagaaggcacccagctctcaccctggcctgacatgtgtgtccctgagcaggttacagtcctctctgagcctctgcttcccatctggaccctgctgggcagggcttctgagctccttagcactagcaggaggggctccaggggccctccctccatggcagccaggacaggactctcaaatgaggacagcagagctcgtggggggctcccacggacccgccgtgggcccaggggaggcagagcctgagccaacagcagtggtgctgtggaccgtggatcctgagggtggcctggggcaagtaccggctgagggtccaggtgggctttgtgtacctttgggtcctggggccctggtgacttggactccaggttagagtcaagtgacaggagaaaggctggtggggccctgtgcttccgacttcatttcgagtgatggcagttcccaggaaggaatccacagctgacggtggctgacagatcagagaatggaaggcgaggcaggcgggcgtctgcgtgacctcaggtgcttggggcccagcagacccagagaaccatttccactaggccagggtgccggaagtgtccacaggtcttagattccctgttcagatgaaaagatttgtgcctttaatgataaaagtgatctgcatagagtcaaaaattcaagccatgggtataaaatgcaagtaaaatccctgccctcacctatcccaccctactacacagagatgtcctctcagctgtaggacgtgcacccaggactcggctcacacatgcggatcctaaggtacctaattgcctagaaaacatgaggatcacccatgtctgcaggtcgactctagaaaacatgaggatcacccatgtctgcagtattcccgggttcattagatcctaaggtacctaattgcctagaaaacatgaggatcacccatgtctgcaggtcgactctagaaaacatgaggatcacccatgtctgcagtattcccgggttcattagatcctaaggtacctaattgcctagaaaacatgaggatcacccatgtctgcaggtcgactccagaaaacatgaggatcacccatgtctgcagtattcccgggttcattagatcctaaggtacctaattgcctagaaaacatgaggatcacccatgtctgcaggtcgactctagaaaacatgaggatcacccatgtctgcagtattcccgggttcattagatcctaaggtacctaattgcctagaaaacatgaggatcacccatgtctgcaggtcgactctagaaaacatgaggatcacccatgtctgcagtattcccgggttcattagatcctaaggtacctaattgcctagaaaacatgaggatcacccatgtctgcaggtcgactccagaaaacatgaggatcacccatgtctgcagtattcccgggttcattagatcctaaggtacctaattgcctagaaaacatgaggatcacccatgtctgcaggtcgactctagaaaacatgaggatcacccatgtctgcagtattcccgggttcattagatcctaaggtacctaattgcctagaaaacatgaggatcacccatgtctgcaggtcgactctagaaaacatgaggatcacccatgtctgcagtattcccgggttcattagatcctaaggtacctaattgcctagaaaacatgaggatcacccatgtctgcaggtcgactccagaaaacatgaggatcacccatgtctgcagtattcccgggttcattagatcctaaggtacctaattgcctagaaaacatgaggatcacccatgtctgcaggtcgactctagaaaacatgaggatcacccatgtctgcagtattcccgggttcattagatcctaaggtacctaattgcctagaaaacatgaggatcacccatgtctgcaggtcgactctagaaaacatgaggatcacccatgtctgcagtattcccgggttcattagatcctaaggtacctaattgcctagaaaacatgaggatcacccatgtctgcaggtcgactccagaaaacatgaggatcacccatgtctgcagtattcccgggttcattagatctgcgcgcgatcgatagatcctaatcaacctctggattacaaaagcatcgtacgcgtacgtgtttggcccccccccctaacgttactggccgaagccgcttggaataaggccggtgtgcgtttgtctatatgttattttccaccatattgccgtcttttggcaatgtgagggcccggaaacctggccctgtcttcttgacgagcattcctaggggtctttcccctctcgccaaaggaatgcaaggtctgttgaatgtcgtgaaggaagcagttcctctggaagcttcttgaagacaaacaacgtctgtagcgaccctttgcaggcagcggaaccccccacctggcgacaggtgcctctgcggccaaaagccacgtgtataagatacacctgcaaaggcggcacaaccccagtgccacgttgtgagttggatagttgtggaaagagtcaaatggctctcctcaagcgtattcaacaaggggctgaaggatgcccagaaggtaccccattgtatgggatctgatctggggcctcggtgcacatgctttacatgtgtttagtcgaggttaaaaaaacgtctaggccccccgaaccacggggacgtggttttcctttgaaaaacacgatgataatatggccacaaccatggtgagcaagggcgaggagctgttcaccggggtggtgcccatcctggtcgagctggacggcgacgtaaacggccacaagttcagcgtgtccggcgagggcgagggcgatgccacctacggcaagctgaccctgaagttcatctgcaccaccggcaagctgcccgtgccctggcccaccctcgtgaccaccctgacctacggcgtgcagtgcttcagccgctaccccgaccacatgaagcagcacgacttcttcaagtccgccatgcccgaaggctacgtccaggagcgcaccatcttcttcaaggacgacggcaactacaagacccgcgccgaggtgaagttcgagggcgacaccctggtgaaccgcatcgagctgaagggcatcgacttcaaggaggacggcaacatcctggggcacaagctggagtacaactacaacagccacaacgtctatatcatggccgacaagcagaagaacggcatcaaggtgaacttcaagatccgccacaacatcgaggacggcagcgtgcagctcgccgaccactaccagcagaacacccccatcggcgacggccccgtgctgctgcccgacaaccactacctgagcacccagtccgccctgagcaaagaccccaacgagaagcgcgatcacatggtcctgctggagttcgtgaccgccgccgggatcactctcggcatggacgagctgtacaagtaacccaaacctaccctgagtttcctagactcactctggaaatttctgtatacacacagaagcttgtgcctctgctcgtgaaggcagagggagggagagctgaagggccagcaccttctcacctgtgggccccctcagtgctcggtcccagagcatgcaggactgtgcctcgtgttcagtttgctggtctgacttcatgctccttgggcaggatatgcatgtgccatgctaggagacatgtggatgtgaagctgggggacaatgtcccctggctatgcctttacaagggaagtaaggaaggtaggaggtgagcctgggagggagggagggaggcgcggagccgccgcaggtgtttcttttactgagtgcagcccatggccgcactcaggttttgcttttcaccttcccatctgtgaaagagtgagcaggaaaaagcaaaa

**Supplementary Figure 8. Genomic sequence of the NEAT1 locus after KI by gRNA-2.** Black, purple, orange, blue, green and red letters indicate the sequences of NEAT1 (partial), 24×MS2, pre-gRNA, IRES, GFP and the CC-rich region, respectively. The yellow highlights indicate the MHAs of the MMEJ-repair. The underline shows the newly fusion gRNA (black) and the corresponding PAM motif (red).
